# Exploring movement patterns and changing distributions of baleen whales in the western North Atlantic using a decade of passive acoustic data

**DOI:** 10.1111/gcb.15191

**Published:** 2020-07-12

**Authors:** Genevieve E. Davis, Mark F. Baumgartner, Peter J. Corkeron, Joel Bell, Catherine Berchok, Julianne M. Bonnell, Jacqueline Bort Thornton, Solange Brault, Gary A. Buchanan, Danielle M. Cholewiak, Christopher W. Clark, Julien Delarue, Leila T. Hatch, Holger Klinck, Scott D. Kraus, Bruce Martin, David K. Mellinger, Hilary Moors‐Murphy, Sharon Nieukirk, Douglas P. Nowacek, Susan E. Parks, Dawn Parry, Nicole Pegg, Andrew J. Read, Aaron N. Rice, Denise Risch, Alyssa Scott, Melissa S. Soldevilla, Kathleen M. Stafford, Joy E. Stanistreet, Erin Summers, Sean Todd, Sofie M. Van Parijs

**Affiliations:** ^1^ NOAA Northeast Fisheries Science Center Woods Hole MA USA; ^2^ University of Massachusetts Boston Boston MA USA; ^3^ Woods Hole Oceanographic Institution Woods Hole MA USA; ^4^ Anderson Cabot Center for Ocean Life New England Aquarium Boston MA USA; ^5^ Naval Facilities Engineering Command Atlantic Norfolk VA USA; ^6^ NOAA Alaska Fisheries Science Center Seattle WA USA; ^7^ Integrated Statistics, Under contract to the NOAA Northeast Fisheries Science Center Woods Hole MA USA; ^8^ New Jersey Department of Environmental Protection Trenton NJ USA; ^9^ Center for Conservation Bioacoustics Cornell Lab of Ornithology Cornell University Ithaca NY USA; ^10^ JASCO Applied Sciences Dartmouth NS Canada; ^11^ NOAA Stellwagen Bank National Marine Sanctuary Scituate MA USA; ^12^ Oregon State University and NOAA Pacific Marine Environmental Laboratory Newport OR USA; ^13^ Fisheries and Oceans Canada Bedford Institute of Oceanography Dartmouth NS Canada; ^14^ Nicholas School of the Environment Duke University Marine Laboratory Beaufort NC USA; ^15^ Pratt School of Engineering Duke University Durham NC USA; ^16^ Syracuse University Syracuse NY USA; ^17^ The Scottish Association for Marine Science (SAMS) Oban UK; ^18^ NOAA Southeast Fisheries Science Center Miami FL USA; ^19^ Applied Physics Laboratory University of Washington Seattle WA USA; ^20^ Maine Department of Marine Resources West Boothbay Harbor ME USA; ^21^ Allied Whale College of the Atlantic Bar Harbor ME USA

**Keywords:** baleen whales, changes in distribution, conservation, North Atlantic Ocean, passive acoustic monitoring, seasonal occurrence

## Abstract

Six baleen whale species are found in the temperate western North Atlantic Ocean, with limited information existing on the distribution and movement patterns for most. There is mounting evidence of distributional shifts in many species, including marine mammals, likely because of climate‐driven changes in ocean temperature and circulation. Previous acoustic studies examined the occurrence of minke (*Balaenoptera acutorostrata*) and North Atlantic right whales (NARW; *Eubalaena glacialis*). This study assesses the acoustic presence of humpback (*Megaptera novaeangliae*), sei (*B. borealis*), fin (*B. physalus*), and blue whales (*B. musculus*) over a decade, based on daily detections of their vocalizations. Data collected from 2004 to 2014 on 281 bottom‐mounted recorders, totaling 35,033 days, were processed using automated detection software and screened for each species' presence. A published study on NARW acoustics revealed significant changes in occurrence patterns between the periods of 2004–2010 and 2011–2014; therefore, these same time periods were examined here. All four species were present from the Southeast United States to Greenland; humpback whales were also present in the Caribbean. All species occurred throughout all regions in the winter, suggesting that baleen whales are widely distributed during these months. Each of the species showed significant changes in acoustic occurrence after 2010. Similar to NARWs, sei whales had higher acoustic occurrence in mid‐Atlantic regions after 2010. Fin, blue, and sei whales were more frequently detected in the northern latitudes of the study area after 2010. Despite this general northward shift, all four species were detected less on the Scotian Shelf area after 2010, matching documented shifts in prey availability in this region. A decade of acoustic observations have shown important distributional changes over the range of baleen whales, mirroring known climatic shifts and identifying new habitats that will require further protection from anthropogenic threats like fixed fishing gear, shipping, and noise pollution.

## Graphical abstract

Limited information exists on the distribution and movement patterns for most baleen whale species in the western North Atlantic Ocean. Previously, we conducted a broad‐scale passive acoustic monitoring study to analyze North Atlantic right whale seasonal distribution and shifts therein, based on identified changes in occurrence patterns starting in 2010. This study uses similar acoustic datasets and protocols to understand the seasonal distribution of humpback, sei, fin, and blue whales within the western North Atlantic, and to determine whether any of these species exhibited similar shifts in distribution patterns across time.

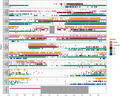

## 1 INTRODUCTION

Seasonal migratory patterns are the foundation of long‐distance movements and dramatic changes in animal distribution for many taxa in the animal kingdom (Dingle, 2014). Many cetaceans undergo long migrations with the purpose of moving from high‐latitude feeding grounds in warmer months, to low‐latitude breeding grounds in colder months (Kellogg, 1929). Baleen whales are among the longest traveled mammals, some covering up to 10,000 km annually (Stevick et al., 2011). Movements are thought to be driven by foraging or social behaviors (e.g., Clapham et al., 1993; Tyack & Whitehead, 1982; Visser, Hartman, Pierce, Valavanis, & Huisman, 2011); however, Corkeron and Connor (1999) also suggested that migration could be influenced by predator avoidance, and highlight that not all whale populations migrate annually (Geijer, Notarbartolo di Sciara, & Panigada, 2016). Non‐migratory populations that remain in tropical and subtropical waters year‐round (Mikhalev, 1997; Širović, Bassett, Johnson, Wiggins, & Hildebrand, 2014) may be supported by year‐round productive foraging grounds (Geijer et al., 2016), as well as reduced energetic expenditure afforded by foregoing long migratory movements (Brown, Corkeron, Hale, Schultz, & Bryden, 1995; Kennedy et al., 2014). Even within migratory populations, some individuals remain on feeding grounds over winter (e.g., Brown et al., 1995; Thomisch et al., 2016; Van Opzeeland, Van Parijs, Kindermann, Burkhardt, & Boebel, 2013). Such intraspecies variation in individual movements are still not well understood, and may be further influenced by differences in gender, age, and reproductive state (Geijer et al., 2016). However, it is clear that baleen whale movement patterns are considerably more complex than previously thought.

Over the last few decades, climate change has led to dramatic increases in ocean temperatures, causing shifts in the distribution of prey species, with foraging animals following suit (Chen, Hill, Ohlemüller, Roy, & Thomas, 2011). The Gulf of Maine, an important feeding ground for many baleen whale species, is one of the fastest warming bodies of water in the world (Pershing et al., 2015), which may influence seasonal shifts in baleen whale presence (Ramp, Delarue, Palsbøll, Sears, & Hammond, 2015) in response to range shifts in prey and fish stocks throughout the western North Atlantic (Nye, Link, Hare, & Overholtz, 2009; Staudinger et al., 2019). North Atlantic right whales (NARWs; *Eubalaena glacialis*), an intensely studied species, are a striking example of these shifts in distributions over the last decade. From 2010 onward, NARWs spent less time in the Gulf of Maine and Bay of Fundy, and more time in mid‐Atlantic waters along the US east coast and the Gulf of St. Lawrence (Davis et al., 2017; Davies et al., 2019). Record et al. (2019) showed that these observed changes in NARW seasonal movements reflect temperature‐driven changes in the distribution of their primary food source, *Calanus finmarchicus*. Additional studies reveal bottom‐up effects of temperature changes, such as shifts in kelp distribution (Merzouk & Johnson, 2011) and collapses of fisheries (Pershing et al., 2015), eventually leading to changes in communities within the entire marine ecosystems (Beaugrand et al., 2019). It is unclear whether other North Atlantic baleen whale species have undergone similar shifts in their movement patterns to NARWs in response to ocean warming and food source redistribution. While the seasonal distribution of humpback whales (*Megaptera novaeangliae*) is relatively well‐known, the movements and distributions of other large baleen whale species (sei, *Balaenoptera borealis*; fin, *B. physalus*; and blue whales, *B. musculus*) throughout the North Atlantic Ocean remain poorly described.

Within the North Atlantic, the humpback whale range extends from breeding grounds in the Caribbean and Cape Verde Islands to feeding grounds off the eastern United States and Canadian seaboard, Iceland, Greenland, and Norway (Hayes, Josephson, Maze‐Foley, & Rosel, 2019; Kennedy et al., 2014). During the spring, summer, and fall, humpback whales in the western North Atlantic are found feeding in the Gulf of Maine, Gulf of St. Lawrence, and in waters off Nova Scotia, Newfoundland, and western Greenland (Katona & Beard, 1990). In winter months, a portion of the North Atlantic humpback whale population visits breeding grounds in the Caribbean and the Cape Verde Islands, and some individuals have even been identified in both breeding grounds (Heenehan et al., 2019; Stevick et al., 2016; Stevick, ØIen, & Mattila, 1998; Wenzel et al., 2009). Passive acoustic data from the western North Atlantic have revealed that humpback whales are present year‐round in the Gulf of Maine (Murray, Rice, & Clark, 2014; Vu et al., 2012), and in winter months off the Scotian Shelf (Kowarski, Evers, Moors‐Murphy, Martin, & Denes, 2018). Tagging studies provided insight on migration between these known coastal feeding and breeding grounds (Kennedy et al., 2014); however, long‐term humpback whale movements among these areas are not well known.

Sei whales are one of the least studied baleen whales, with most information on their distribution derived from historic whaling records, stranding records, and visual surveys (COSEWIC, 2003; Hayes et al., 2019; Mead, 1977). In the western North Atlantic, their range extends from mid‐ to low‐ latitudes to as far north as Labrador (Kapel, 1985; Kellogg, 1929; Olsen et al., 2009; Prieto, Silva, Waring, & Gonçalves, 2014) and the Davis Strait (Mitchell, 1974). The southern limit of their range remains unknown; however, stranding reports document sei whales as far south as Florida (Miller, 1924) and Mexico (Miller, 1928). Migratory movements of sei whales in the western North Atlantic are not yet well understood, but they are believed to move northward in June and July from southern New England to eastern Canada (Mitchell, 1975), and move southward in September and October (CETAP, 1982). During the spring and summer, sei whales are sighted in northern portions of the US Atlantic Exclusive Economic Zone (EEZ), including Georges Bank, the Gulf of Maine, and south of New England (Halpin et al., 2009). Often found in the deeper waters off the continental shelf edge, including the Scotian Shelf edge during the spring feeding season (Hain, Hyman, Kenney, & Winn, 1985), sei whales are also seen in shallower waters of the continental shelf in the Great South Channel and Massachusetts Bay (Halpin et al., 2009; Payne et al., 1990). Recently, satellite tag studies revealed westward movements of tagged individuals from the Azores to the Labrador Sea in the summer (Olsen et al., 2009; Prieto et al., 2014). Few studies have documented North Atlantic sei whale vocalizations, until recent work recorded and described sei whale vocalizations off New England and the Azores (Baumgartner et al., 2008; Romagosa, Boisseau, Cucknell, Moscrop, & McLanaghan, 2015; Tremblay, Van Parijs, & Cholewiak, 2019).

Fin whales are frequently observed in the western North Atlantic, from Cape Hatteras, North Carolina to Greenland (Edwards, Hall, Moore, Sheredy, & Redfern, 2015). A global review of fin whale sightings and acoustic data showed year‐round presence throughout most of the US EEZ, commonly occurring in the Gulf of Maine and in Canadian waters off Nova Scotia (Edwards et al., 2015; Hain, Ratnaswamy, Kenney, & Winn, 1992). Acoustic records revealed the year‐round presence of fin whales in Massachusetts Bay and the New York Bight (Morano et al., 2012; Muirhead et al., 2018), as well as occurrence from September through June in offshore waters surrounding Bermuda and the Mid‐Atlantic Ridge (Clark & Gagnon, 2004; Nieukirk et al., 2012; Nieukirk, Stafford, Mellinger, Dziak, & Fox, 2004; Watkins, Tyack, Moore, & Bird, 1987). While New England waters provide important feeding grounds, mating and calving grounds remain unknown. Hain et al. (1992) suggest US mid‐Atlantic latitudes for calving grounds based on neonatal stranding analyses, but this has not been confirmed by at‐sea surveys. While fin whales do undergo seasonal movements (Silva et al., 2011), their broad‐scale distribution year‐round suggests the possibility that they do not undergo the same large‐scale migrations in the North Atlantic as other baleen whales, similar to fin whales in the North Pacific (Oleson, Sirovic, Bayless, & Hildebrand, 2014).

In the western North Atlantic, blue whales are mainly sighted off eastern Canada, with occasional sightings in the Gulf of Maine (Wenzel, Mattila, & Clapham, 1988) and other waters within the US EEZ (CETAP, 1982). The northern part of their range includes waters off Nova Scotia, Newfoundland, and Labrador, and extends as far north as the Davis Strait (Jonsgård, 1955; Moors‐Murphy et al., 2019). From spring through summer, blue whales occur predominantly in the Gulf of St. Lawrence, where the population is well‐studied (Sears et al., 1990). In winter months, blue whales are found from southern Newfoundland to the Davis Strait (Mansfield, 1985), while acoustic detections also indicate their presence as far south as the New York Bight and near the Mid‐Atlantic Ridge (Muirhead et al., 2018; Nieukirk et al., 2004). They are seen and heard year‐round outside the Gulf of St. Lawrence in waters off Nova Scotia (Moors‐Murphy et al., 2019). While their southern range limit is unknown, acoustic detections of blue whales have occurred in deep water north of the West Indies and east of the US EEZ (Clark, 1995; Nieukirk et al., 2004). There have been a few historical strandings in the Caribbean (Harmer, 1923) and the Gulf of Mexico (Baughman, 1946), supporting suggestions that their range extends at least that far south (Yochem & Leatherwood, 1985). Their tendency to use deeper, rather than coastal waters makes their seasonal movements difficult to study. However, satellite tag studies show movements of blue whales from the Gulf of St. Lawrence to North Carolina, including both on‐ and off‐shelf waters, extending to deeper waters around the New England Seamounts (Lesage, Gavrilchuk, Andrews, & Sears, 2017).

Passive acoustic monitoring (PAM) provides robust data to explore multiple species' simultaneous occurrence across seasons. Decadal studies using PAM have monitored seasonal distributions of fin whales (Nieukirk et al., 2012); tracked migratory movements of humpback whales (Abileah, Martin, Lewis, & Gisiner, 1996) and blue whales (Stafford, Nieukirk, & Fox, 1999); and provided new information on movements for minke whales (*Balaenoptera acutorostrata*; Risch et al., 2014) and NARWs (Davis et al., 2017). Within the North Atlantic, well‐known songs or call types are unequivocally attributed to each of the species discussed in this paper and are widely used to assess their presence. Here we use patterned song notes and other sounds produced by humpback whales (Payne & McVay, 1971; Stimpert, Au, Parks, Hurst, & Wiley, 2011), downsweeps produced by sei whales (Baumgartner et al., 2008), 20 Hz pulses produced by fin whales (Watkins et al., 1987), and song notes produced by blue whales (Mellinger & Clark, 2003) to examine large‐scale species distribution. Most of these signals are sex‐specific (humpback whale song: Winn & Winn, 1978; fin whale 20 Hz pulses: Croll et al., 2002), and often seasonal (blue whale song: Moore, Stafford, Mellinger, & Hildebrand, 2006; Stafford, Mellinger, Moore, & Fox, 2007). Although we will miss species' presence when they use other call types or are silent, we can still capture large‐scale distribution patterns throughout the periods that they use these known vocalizations.

Previously, we conducted a broad‐scale PAM study across the western North Atlantic to analyze NARW seasonal distribution (Davis et al., 2017). Based on identified changes in occurrence patterns starting in 2010, we found NARW acoustic detections significantly decreased in the Gulf of Maine region, and increased in mid‐Atlantic regions of the US eastern seaboard. Here, we use similar acoustic datasets and protocols to understand the seasonal distribution of humpback, sei, fin, and blue whales within the western North Atlantic Ocean, and to determine whether any of these species exhibited similar shifts in distribution patterns across time.

## 2 MATERIALS AND METHODS

### 2.1 Data collection

**FIGURE 1:**
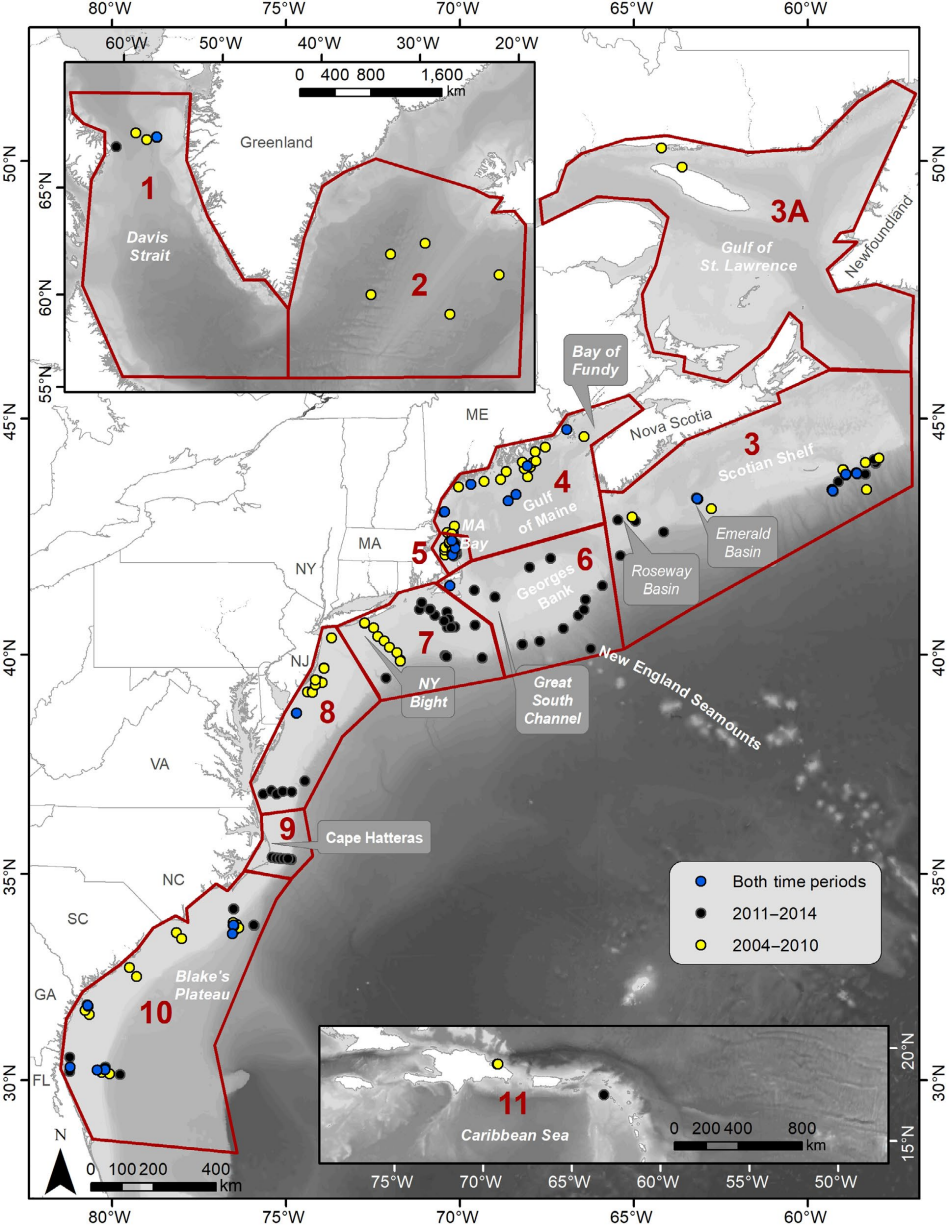
Locations of available passive acoustic recorders used for this study extending from the Caribbean (bottom right map inset) to the northernmost locations in the Davis Strait (top left map inset). Yellow points indicate the locations of recorders available from 2004 to 2010; black points indicate the locations of recorders available from 2011 to 2014; and blue points indicate locations of recorders available for any amount of time across both time periods. Red boundaries outline the designated regions, which were defined following the methods in Davis et al. (2017). Region numbers correspond to the following geographic areas: 1. Davis Strait; 2. Eastern Greenland; 3. Scotian Shelf; 3A. Gulf of St. Lawrence; 4. Gulf of Maine; 5. Massachusetts Bay; 6. Georges Bank; 7. Southern New England and New York Bight; 8. Mid‐Atlantic; 9. Cape Hatteras, North Carolina; 10. Southeast United States; 11. Caribbean

All available passive acoustic recordings from over 100 research projects throughout the western North Atlantic Ocean were combined to create a decade‐long dataset. A total of 35,033 recording days of data were collected from 2004 to 2014 from 281 passive acoustic recorders deployed between Saba in the Caribbean and the Davis Strait off western Greenland (Figure 1). Most recording sites were located on the continental shelf or along the shelf edge with only six sites in off‐shelf (off eastern Greenland [region 2] and a New England Seamount [Bear Seamount, region 6]) waters; therefore, this analysis was largely restricted to the continental shelf and shelf break region. The dataset was broken up into 11 geographic regions, based on acoustic data availability and biologically relevant areas (Figure 1; Davis et al., 2017). The Gulf of St. Lawrence was designated as a separate subregion (region 3A) to reflect its biological importance (Meyer‐Gutbrod, Greene, & Davies, 2018); however, only 2 months of recordings were made available for our study in this region, so the Gulf of St. Lawrence (region 3A) was combined with the Scotian Shelf (region 3) and incorporated in the results as one region (region 3) for all analyses.

Recordings were collected using five different types of bottom‐mounted passive acoustic recorders (Table 1) as follows: the High‐frequency Acoustic Recording Package (HARP; Wiggins & Hildebrand, 2007), the Marine Autonomous Recording Unit (MARU; Clark, Brown, & Corkeron, 2010), the Autonomous Multichannel Acoustic Recorder (AMAR; Moloney, Hillis, Mouy, Urazghildiiev, & Dakin, 2014), the Autonomous Underwater Hydrophone (AUH; Fox, Matsumoto, & Lau, 2001), and the Guardbuoy (Akoostix Inc/Geospectrum Technologies; http://geospectrum.ca). Data were collected from 281 recorders, ranging from a minimum of 25 days to a maximum of 2 years (Table 1). Of these recorders, 56 used a duty cycled recording schedule, recording 12%–95% of the time, and 225 recorded continuously. The majority of recordings (206 out of 281) were sampled at 2 kHz, with some ranging up to 250 kHz. All recordings were low‐pass filtered and decimated to 2 kHz to ensure comparability and analytical consistency across datasets. Recordings were further resampled to 120 Hz for adequate analyses of lower‐frequency signals, in this case, vocalizations of fin and blue whales.

**TABLE 1:** Summary of recorder locations, types, and configurations for all analyzed data. Recorders are grouped by region and deployment location with data gaps indicating periods of time within the recording dates that no recorders were in the water in that area

Location	Region	Unit type	No. units	Configuration	Recording dates	Central latitude	Central longitude	Average depth (m)	Recording schedule (minutes on/off)	Data gaps
Davis Strait	1	AUH	3	Line	10/2006–10/2007	67.10	−57.72	350	Continuous	
Davis Strait	1	AUH	2	Single	11/2011–09/2013	61.50	−58.75	350	15/45 (25%)	
Eastern Greenland	2	AUH	5	Array	05/2007–07/2008	60.90	−29.30	2,470	Continuous	
Gulf of St. Lawrence	3A	MARU	2	Single	06/2010–09/2010	50.07	−63.92	30	Continuous	
Emerald Bank, Scotian Shelf	3	AUH	2	Single	08/2005–06/2006	43.15	−63.67	129	Continuous	
Roseway Basin, Scotian Shelf	3	AUH	1	Single	08/2005–06/2006	42.97	−65.06	155	Continuous	
Eastern Scotian Slope	3	MARU	1–2	Single	07/2006–01/2007	43.78	−58.74	1,548	10/50 (17%)	09/2006–12/2006
Eastern Scotian Slope	3	MARU	2–5	Single	08/2007–03/2009	43.99	−58.40	1,573	7/53 (12%)	10/2007–12/2007, 03/2008–06/2008, 09/2008–12/2008
Eastern Scotian Slope	3	AMAR	1	Single	03/2010	43.95	−59.00	1,100	Continuous	
Eastern Scotian Slope^a^	3	AMAR	3	Single	10/2012–09/2013	43.94	−58.52	1,578	13/2 (87%)	04/2013
Emerald Basin, Scotian Shelf	3	Guardbuoy	1	Single	08/2013	43.37	−63.22	170	4.75/0.25 (95%)	
Roseway Basin, Scotian Shelf	3	Guardbuoy	1–2	Single	08/2013–09/2013	42.90	−65.22	150	4.75/0.25 (95%)	
Eastern Scotian Slope	3	AMAR	3	Single	11/2013–10/2014	43.95	−58.54	1,493	17/3 (85%)	04/2014
Brown's Bank	3	MARU	1	Single	04/2014–09/2014	42.15	−65.39	432	Continuous	
Brown's Bank	3	AUH	1	Single	06/2014–10/2014	42.65	−64.15	750	Continuous	
Eastern Scotian Slope	3	AMAR	2	Single	07/2014–10/2014	43.60	−59.20	820	11.3/3.7 (75%)	
Bay of Fundy	4	MARU	1	Single	08/2004, 08/2005	44.63	−66.44	194	Continuous	
Gulf of Maine	4	MARU	7	Single	07/2008–10/2008	44.02	−68.03	112	Continuous	
Gulf of Maine	4	MARU	1	Single	07/2009–10/2009	44.04	−68.07	35	Continuous	
Gulf of Maine^a^	4	MARU	1	Single	10/2009–10/2010	43.30	−68.62	168	Continuous	06/2010
Gulf of Maine	4	MARU	2	Single	07/2010–01/2011	43.08	−70.46	80	Continuous	
Gulf of Maine	4	MARU	9	Single	09/2010–12/2010	44.04	−68.57	75	Continuous	
Gulf of Maine	4	MARU	2	Single	10/2010–05/2011	43.37	−68.51	168	Continuous	
Gulf of Maine	4	MARU	1	Single	07/2011–09/2011	44.01	−68.07	85	Continuous	
Massachusetts Bay	5	MARU	1	Single	01/2006–05/2010	42.42	−70.28	58	Continuous	06/2006, 06/2008
Massachusetts Bay	5	MARU	3	Single	04/2011–05/2011	42.21	−70.17	44	Continuous	
Massachusetts Bay	5	MARU	2	Single	08/2011–10/2011	42.20	−70.15	76	Continuous	
Massachusetts Bay^a^	5	MARU	1–2	Single	06/2013–04/2014	42.25	−70.42	72	Continuous	
Massachusetts Bay	5	MARU	1	Single	10/2014–01/2015	42.40	−70.13	78	Continuous	
Nantucket Sound	6	AMAR	1	Single	10/2010–07/2011	41.50	−70.30	16	Continuous	
Georges Bank	6	MARU	3	Single	03/2012–06/2012	41.52	−68.87	88	Continuous	
Georges Bank	6	MARU	1	Single	03/2012–04/2012	42.09	−67.40	64	Continuous	
Georges Bank^a^	6	MARU	1	Single	03/2012–09/2014	40.40	−66.520	333	Continuous	07/2012–04/2014
Georges Bank	6	MARU	1	Single	03/2012–09/2014	41.36	−66.160	225	Continuous	05/2012–04/2014
Georges Bank	6	MARU	1	Single	05/2013–09/2014	40.23	−68.220	338	Continuous	08/2013–04/2014
Georges Bank	6	MARU	1	Single	04/2014–09/2014	40.58	−67.04	375	Continuous	
Georges Bank^a^	6	AUH	1	Single	06/2014–01/2015	40.13	−66.25	3,500	Continuous	
Bear Seamount	6	AMAR	1	Single	07/2014–01/2015	40.29	−67.72	800	5.6/24.3 (19%)	
New York^b^	7	MARU	6–7	Line	02/2008–03/2009	40.32	−72.22	77	Continuous	05/2008–08/2008
Southern New England^a^, ^b^	7	MARU	5–8	Line	11/2011–02/2014	40.81	−70.52	50	Continuous	10/2012–02/2013
Georges Bank	7	MARU	1	Single	05/2013–04/2014	40.65	−69.58	48	Continuous	08/2013–04/2014
Georges Bank	7	MARU	1–3	Single	05/2013–09/2014	39.93	−70.16	334	Continuous	07/2013–04/2014
Georges Bank	7	MARU	1	Single	04/2014–09/2014	39.49	−72.13	353	Continuous	
New York Harbor	8	MARU	1	Single	02/2008–03/2009	40.37	−73.70	27	Continuous	05/2008–08/2008
New Jersey	8	MARU	1–4	Single	03/2008–11/2009	39.44	−74.08	24	Continuous	06/2009–08/2009
New Jersey	8	MARU	2	Single	06/2008–09/2008	39.42	−74.08	24	5/25 (17%)	
Delaware	8	AMAR	1	Single	06/2010–08/2011	38.70	−74.70	21	Continuous	
Virginia^a^	8	MARU	4–5	Single	06/2012–07/2014	36.90	−75.26	33	Continuous	
Virginia	8	HARP	1	Single	06/2014–01/2015	37.17	−74.47	982	Continuous	
Cape Hatteras^a^	9	HARP	1	Single	03/2012–11/2014	35.34	−74.86	935	Continuous	04/2012–10/2012, 03/2013–05/2014
Cape Hatteras^b^	9	MARU	4–5	Line	10/2013–01/2015	35.37	−75.16	41	Continuous	06/2014–10/2014
North Carolina	10	MARU	2	Line	01/2006–04/2006	33.61	−78.15	22	Continuous	
South Carolina	10	MARU	2	Line	01/2006–04/2006	32.66	−79.40	21	Continuous	
Georgia	10	MARU	2	Line	01/2006–04/2006	31.78	−80.84	18	Continuous	
North Carolina	10	HARP	1–2	Single	10/2007–06/2013	33.77	−76.29	475	5/5 (50%)	01/2008–05/2008, 09/2008–04/2009, 08/2009–07/2010, 03/2011–08/2011, 12/2011–07/2012
North Carolina	10	HARP	1–2	Single	11/2009–04/2010	33.74	−76.50	253	5/10 (33%)	
North Carolina	10	MARU	2	Array	07/2008	33.80	−76.45	233	Continuous	
Florida	10	HARP	1–2	Single	04/2009–07/2011	30.27	−80.32	63	5/10 (33%)	12/2009–02/2010
Florida	10	MARU	2	Array	09/2009–01/2010	30.18	−80.20	204	Continuous	10/2009–12/2009
Georgia	10	MARU	1	Single	11/2009–06/2011	31.83	−80.70	16	Continuous	05/2010–01/2011
Florida^b^	10	MARU	1	Single	11/2009–05/2014	30.34	−81.21	17	Continuous	05/2010–01/2011, 06/2011–12/2011, 05/2012–12/2012, 05/2013–11/2013
Georgia^a^	10	MARU	1	Single	06/2012–04/2013	31.86	−80.72	18	Continuous	
North Carolina	10	MARU	1	Single	06/2012–04/2013	34.17	−76.51	34	Continuous	
Georgia	10	MARU	1	Single	11/2012–05/2014	30.57	−81.23	14	Continuous	04/2013–11/2013
Florida	10	HARP	1	Single	05/2013–01/2015	30.27	−80.06	806	Continuous	06/2013–02/2014
Samana, Dominican Republic	11	MARU	1	Single	01/2009–03/2009	19.16	−69.18	29	Continuous	
Saba Bank, Caribbean	11	MARU	1	Single	10/2011–04/2012	17.51	−63.19	30	30/90 (25%)	

Abbreviations: AMAR, Autonomous Multichannel Acoustic Recorder; AUH, Autonomous Underwater Hydrophone; HARP, High‐frequency Acoustic Recording Package; MARU, Marine Autonomous Recording Unit.

^a^ Recording unit from this deployment used for logistic regression analysis to determine number of detections/hr needed for fin whale presence evaluation.

^b^ Recording unit from this deployment used for missed detection rate analysis.

Acoustic detection ranges can vary significantly depending on the recording equipment, location, whale or recorder depth, bathymetry and environmental conditions, as well as by signal type and behavioral context (Cholewiak et al., 2018; Širović, Hildebrand, & Wiggins, 2007; Stafford et al., 2007). Previous acoustic studies examined detection ranges over which the species‐specific vocalizations used in this study can be heard in varying oceanographic conditions (Baumgartner et al., 2008; Cholewiak et al., 2018; Kowarski et al., 2018; Širović et al., 2007; Stafford et al., 2007), in some cases for the same datasets used in this study. We used results from these studies here as guidelines for the distance over which each species may be detected within this study's geographic range (see Table 2). Taking this information into account and to be conservative, only a single recorder was selected for analysis when recorders were congregated in groups or arrays with units spaced at 20 km or less; this approach minimized duplicate detections across receivers as best as possible. Acoustic analyses were focused on data collected between January 2006 and December 2014, with the exception of additional data collected in 2004 and 2005 in the Bay of Fundy, Emerald Basin, and Roseway Basin, Canada, as these were the only long‐term recordings available for these areas.

**TABLE 2:** Detection ranges found from previous studies for each species, in varying water depths. Letters next to species names indicate water depth category (D, deep [>1,000 m]; M, medium [100–1,000 m]; S, shallow [<100 m]). For each species, the frequency band, water depth in meters, study location, detection ranges and source level are listed

Species	Frequency band (Hz)	Water depth	Study location	Detection range (km)	Source level (dB re 1 μPa)	Reference
Humpback (S)	36–355	Shallow (30−100 m)	Massachusetts Bay, North Atlantic	5–30	167	Cholewiak et al. (2018)
Humpback (D)	20–1,800	Deep (1,500 m)	Scotian Shelf, North Atlantic	1–53, up to 100	162	Kowarski et al. (2018)
Sei (M)	34–82	Medium (100–192 m)	Great South Channel, North Atlantic	10–15, up to 20	156	Baumgartner et al. (2008)
Fin (S)	18–22	Shallow (30–100 m)	Massachusetts Bay, North Atlantic	30	180	Cholewiak et al. (2018)
Fin (M)	25	Medium (340–450 m)	Gulf of Alaska, North Pacific	10–100	171	Stafford et al. (2007)
Fin (D)	15–28	Deep (3,000 m)	Southern Ocean	56	189	Širović, Hildebrand, and Wiggins (2007)
Blue (M)	16–20	Medium (340–450 m)	Gulf of Alaska, North Pacific	10–105, up to 195	180	Stafford et al. (2007)
Blue (D)	25–29	Deep (3,000 m)	Southern Ocean	25–200	189	Širović et al. (2007)

### 2.2 Detection and classification of calls

All acoustic data were processed using the Low Frequency Detection and Classification System (LFDCS; Baumgartner & Mussoline, 2011), which creates conditioned spectrograms using a short‐time Fourier transform with a data frame of 512 samples and 75% overlap (80% overlap for the 120 Hz decimated data), resulting in a time step of 64 ms and frequency resolution of 3.9 Hz (for 120 Hz data: 853 ms time step and 0.23 Hz frequency resolution). After tracing contour lines, or “pitch tracks,” through tonal sounds, the program uses multivariate discriminant function analysis to classify the pitch tracks into species‐specific call types based on a call library. Each detection is assigned a Mahalanobis distance (MD), which measures the deviation of a sound's pitch track from the assigned call type (see Baumgartner & Mussoline, 2011 for a more complete description). A lower MD indicates a closer match to the assigned call type. For a well‐developed call type in the LFDCS (i.e., the seven attributes used in the discriminant function analysis are multivariate normal), 75% of pitch‐tracks for the call type will have an MD of 3.0 or less (Baumgartner et al., 2013). Setting an MD threshold is necessary to minimize the false detection rates, but in doing so causes some true detections to be missed in the analysis. The MD threshold of 3.0 was chosen for all call types detected and classified in the humpback, sei, and fin whale call library. However, for blue whales, false detection rates were lower than any of the other species, thus an MD of 5.0 was chosen to decrease the probability of missing true detections.

All vocalizations were classified based on a user‐developed call library (expanded from Davis et al., 2017; Table S1); our library for the 2 kHz sampled data included two of our target species—humpback and sei whales. Given the low frequency characteristics of fin and blue whale vocalizations, an additional call library was created for these two species that matched the decimated 120 Hz sampled data.

All LFDCS detections were manually reviewed by a number of trained acoustic analysts to determine daily presence of each of the four baleen whale species. A true detection was defined as a pitch track that correctly classified a call or song unit to the species that produced it (Bonnell et al., 2016). Given the variability of each species' vocalizations, the specific methodology to determine daily acoustic presence was different for each species. That process is described in more detail below.

### 2.3 Baleen whale call types used for detection and classification

**FIGURE 2:**
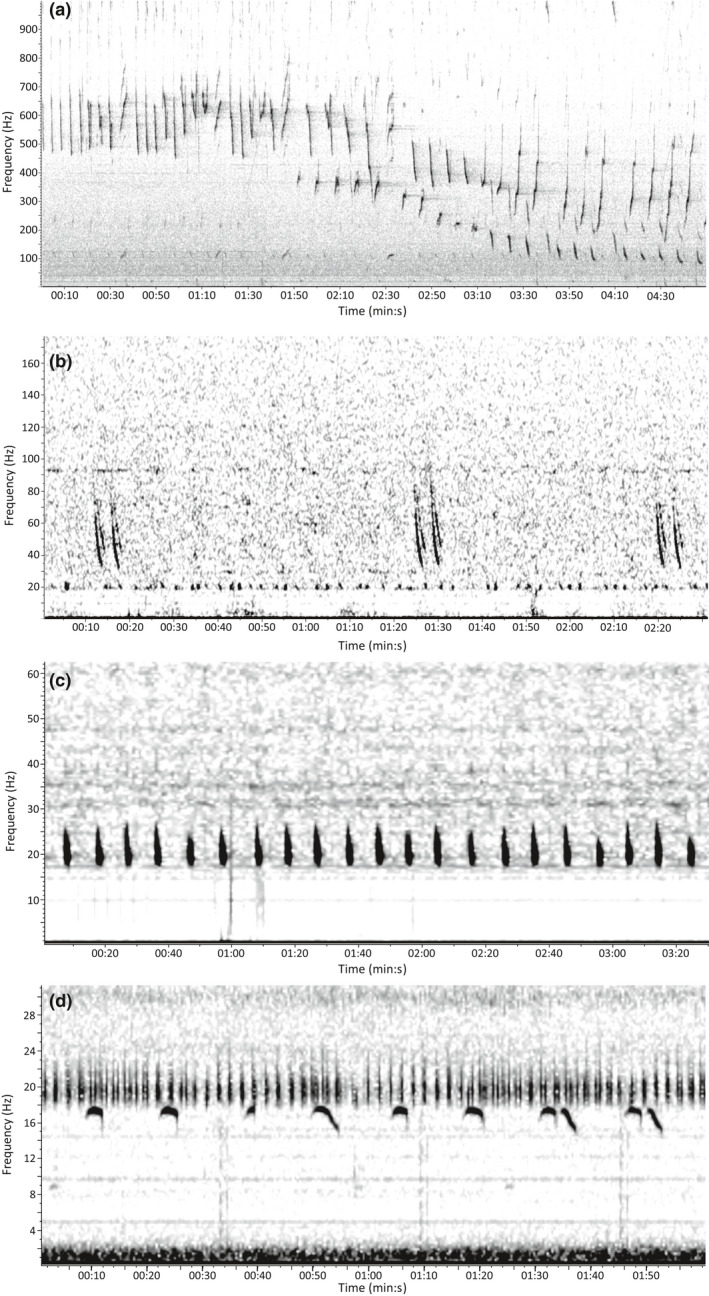
Spectrogram examples of (a) humpback whale song, (b) sei whale doublet downsweeps, (c) fin whale 20 Hz pulses, and (d) blue whale A, B, and AB song notes

Humpback whale males produce complex song that changes annually (Payne & McVay, 1971; Payne & Payne, 1985; Winn & Winn, 1978), and has been recorded throughout their entire range and across seasons (Figure 2a; Clark & Clapham, 2004; Kowarski et al., 2018; Mattila, Guinee, & Mayo, 1987; Vu et al., 2012). Non‐song vocalizations, or social sounds, vary with some calls being similar to those found in song while others are completely different. These non‐song vocalizations are produced by both sexes and across ages (Dunlop, Cato, & Noad, 2008; Fournet, Jacobsen, Gabriele, Mellinger, & Klinck, 2018; Stimpert, 2010; Stimpert et al., 2011). Given that humpback whale song can be highly variable between years, the call library described in Baumgartner and Mussoline (2011) was expanded and improved for this analysis to include a wider variety of examples of humpback whale vocalizations, across all years, to increase detection probability (Table S1). While the call library expansion focused on capturing song notes, the detector's versatility also reliably detected some social sounds, due to their similarity to some song notes. Therefore, all humpback whale detections (song and social sounds) with an MD of 3.0 or less were screened for daily presence. Any detection that was correctly identified to species was considered a true detection. A day was then marked as present for humpback whales if one true detection was found within at least three humpback whale vocalizations, occurring over a 10 min window. The 10 min window was deemed sufficient to clearly distinguish putative humpback whale vocalizations from those of other species.

Sei whales in the North Atlantic produce low‐frequency downsweeps (Figure 2b), from 82 to 34 Hz, as single, doublet, triplet, or more series of vocalizations (Baumgartner et al., 2008). These downsweeps can also be found associated with other newly reported call types thought to represent song (Tremblay et al., 2019). It is currently unknown whether these vocalizations are sex‐biased, or how they may vary regionally and seasonally. The LFDCS call library described in Baumgartner and Mussoline (2011) contains the 82–34 Hz sei whale downsweep, which was the call type we focused on in this study. All sei whale downsweep detections with an MD of 3.0 or less were manually screened for the daily presence of a doublet or triplet (following the same methods as described by Baumgartner et al. (2008); doublets and triplets were defined as two or three repeated downsweeps, respectively, with roughly 3.5 s elapsed between the start of successive calls). Sei whales were considered present if a true detection (at least one downsweep detected within a doublet or triplet) was found for that day. As single sei whale downsweeps can resemble some vocalizations produced by fin and blue whales (Berchok, Bradley, & Gabrielson, 2006; Širović, Hildebrand, & Thiele, 2006), only the occurrence of the downsweeps as doublets or triplets were selected to ensure confidence in species identification.

Fin whales produce 20 Hz pulses, occurring in 7–19 s intervals, with bouts lasting up to 32.5 hr (Figure 2c; Julien Delarue, personal communication; Morano et al., 2012; Watkins et al., 1987). These vocalizations are thought to be produced solely by males as a breeding display (Croll et al., 2002). They have been documented throughout the year in their western North Atlantic range, and thus are excellent indicators of male fin whale presence. A call library for fin whale 20 Hz pulses was built for the data sampled at 120 Hz. To validate this call library, a full year of data from nine sites (18 recorders total) were selected across the dataset range (marked with “a” in Table 1). These data were examined every third hour of each day on the first, 11th, and 21st day of the month to look for fin whale presence. These hours were manually verified for true detections with an MD of 3.0 or less. Using the methods described in Baumgartner and Mussoline (2011), a logistic regression was applied to these results to facilitate reducing the size of the dataset that ultimately needed to be manually verified for confident species detection. This analysis revealed that a minimum number of 29 detections per hour need to be detected to ensure that a fin whale was truly detected in that hour with a confidence of 90%. To confirm true fin whale presence in the full dataset of 281 recorders, all hours with at least 29 detections (as determined by the logistic regression above) were then manually verified for daily presence of fin whale 20 Hz pulses. From those hours with 29 or more detections, fin whales were considered present for that day if a true detection was found within a regular interpulse interval pattern of at least three other 20 Hz pulses. Furthermore, to ensure accurate representation of fin whale presence in duty cycled data, all detections for all hours of recorders that had a recording duty cycle of 30% of the time or less were manually reviewed for accurate daily presence. This accounted for 21 recorders, or 7% of the data where all hours were manually verified (see Table 1 for a summary of these decisions).

The most common vocalizations documented from blue whales in the North Atlantic are their low frequency song, which is made up of repeated phrases, comprised of song notes, with 1–2 min intervals (Mellinger & Clark, 2003), thought to be produced by males (Oleson et al., 2007). A call library for blue whales was built for the data sampled at 120 Hz, and created for A, B, and AB phrases (as described by Mellinger & Clark, 2003; Figure 2d, Table S1). All detections with an MD of 5.0 or less were manually screened. Daily presence for blue whales was confirmed if there were three song phrases visible, including at least one true detection. The low frequency band in which blue whale song occurs is often overlapped with boat or background noise and in noisy situations it can be difficult to identify song units with confidence. Only accepting detections when three or more phrases occurred ensured our confidence in the presence of the blue whale song.

### 2.4 Validation of LFDCS performance

The manual verification of each detection ensured a 0% false detection rate in daily presence. To evaluate the missed detection rate of the LFDCS for each of the four species, three regions (Southern New England, Cape Hatteras, and Southeast United States; regions 7, 9, and 10; see Figure 1) were chosen for manual analysis of the recorded audio. Owing to the large size of the dataset, all regions and recorders could not be included. These regions were selected to incorporate variability across the datasets' geographic, water depth, and temporal range, using one recorder type (MARU) for a comparable assessment. When available, a full year of data from one recording site was taken from the two time periods compared in this analysis (before and after 2010) for regions 7 and 10, and data from the only available time period (after 2010) in region 9 were taken (marked with “b” in Table 1).

Every fifth day was manually screened by a trained acoustic analyst for the daily presence of each call type described above for each of the species. Long‐term spectral averages (LTSAs) were viewed using the MATLAB‐ (Mathworks) based custom sound analysis software program Triton (Wiggins & Hildebrand, 2007). When further inspection was needed, the sound analysis software Raven Pro 1.5 (Bioacoustics Research Program, 2014) was used to examine the spectrogram in more detail; thus allowing a more accurate assessment of the presence or absence of certain vocalization types. When the vocalizations of a given species were observed, that day was marked as positive for presence of that species. The number of days of each species' presence found by the manual screening of acoustic data was compared to the days marked as present using confirmed detections from LFDCS. Missed detection rates were calculated using the confusion matrix method as described in Baumgartner et al. (2019).

### 2.5 Review and analysis of call detections

Daily presence of all call types for each of the four species was summarized into weekly bins and plotted across the spatial extent of the passive acoustic recorders (regions 1–11) over (a) the entire time series (2004–2014); and (b) the time series split between 2004 to 2010 and 2011 to 2014. This split was the same as used for the analysis of NARW acoustic presence in Davis et al. (2017), which was based on the timing of the marked climatological shifts in the Gulf of Maine (Record et al., 2019) and multiple species' distribution changes in the western North Atlantic Ocean (Pershing, Mills, Dayton, Franklin, & Kennedy, 2018). Only regions with acoustic occurrence in both time periods were compared.

We ran a generalized linear model (GLM) in R 3.4.1 (R Core Team, 2017), using the libraries *MASS* (Venables & Ripley, 2002), *car* (Fox & Weisburg, 2011), and *phia* (De Rosario‐Martinez, 2015) to test whether the annual occurrence of each species across regions differed over the two time periods. In this analysis, we defined the number of days per year (summed across all recorders for each region) with detected species‐specific vocalizations as the dependent variable, and defined time periods (2004–2010; 2011–2014) and regions as independent variables, with their interaction effects included in the model. A GLM with a Poisson distribution with log‐link was run given that the detection data were counts, accounting for zero‐inflated, discrete data. Within each year and region, the number of recording days was multiplied by the duty‐cycle to correct for non‐continuous data. As recording effort (the number of days during which recorders were present) varied across time and region, we included the log of the number of days during which recorders were present plus 1 (because for some time*region cells, there were no recorders present) as an offset in the model. This procedure resulted in the following model structure:

nDaysWithWhales∼timePeriod∗Region,family='poisson',

Offset=log(nDaysRecording+1).

Lastly, results from these analyses were compared to the NARW's daily presence data from Davis et al. (2017) to compare the seasonal presence of five baleen whale species.

## 3 RESULTS

**FIGURE 3:**
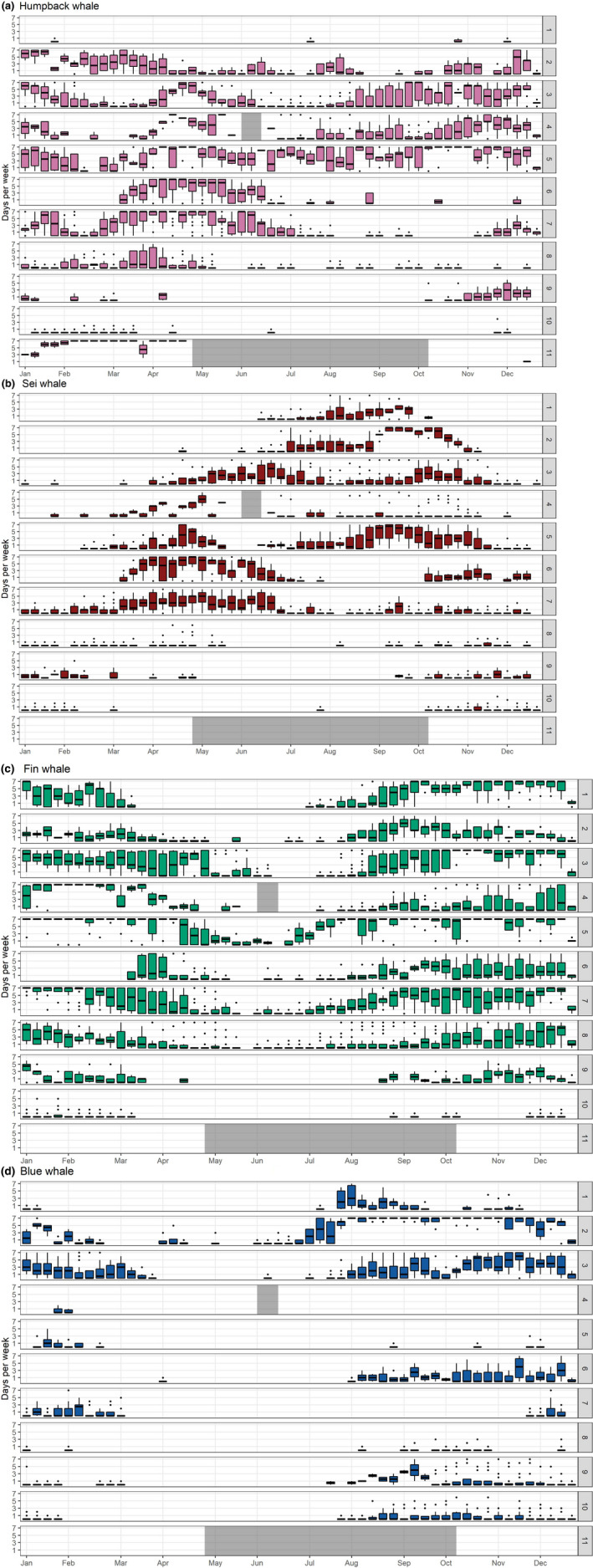
Weekly presence summary: Boxplots representing the average number of days per calendar week per recording site with confirmed acoustic presence for (a) humpback whales; (b) sei whales; (c) fin whales, and (d) blue whales, across all recorders in each region described in Figure 1 and for all years of the study (2004–2014). Horizontal lines within the boxes indicate the median, box boundaries indicate the 25th (lower boundary) and 75th (upper boundary) percentiles, vertical lines indicate the largest (upper whisker) and smallest (lower whisker) values no further than 1.5 times the interquartile range, and black dots represent outliers. Grey blocks indicate weeks where no data were available for that region

**FIGURE 4:**
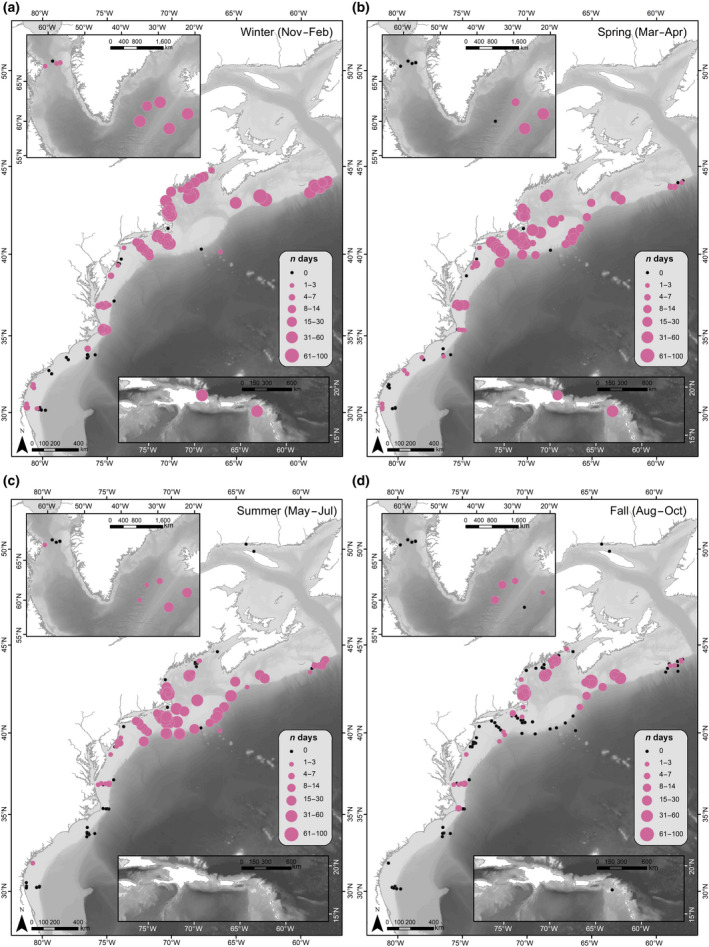
Humpback whale seasonal occurrence maps: The number of days per season with confirmed North Atlantic humpback whale acoustic detections, summarized for all available recording locations (2004–2014). Filled pink circles indicate humpback whale acoustic presence, and circle size indicates the number of days with humpback whale acoustic detections during a season. Black dots indicate recorder locations with no humpback whale acoustic presence for any year during that season (defined as: (a) Winter [November–February]; (b) Spring [March–April]; (c) Summer [May–July]; and (d) Fall [August–October])

**FIGURE 5:**
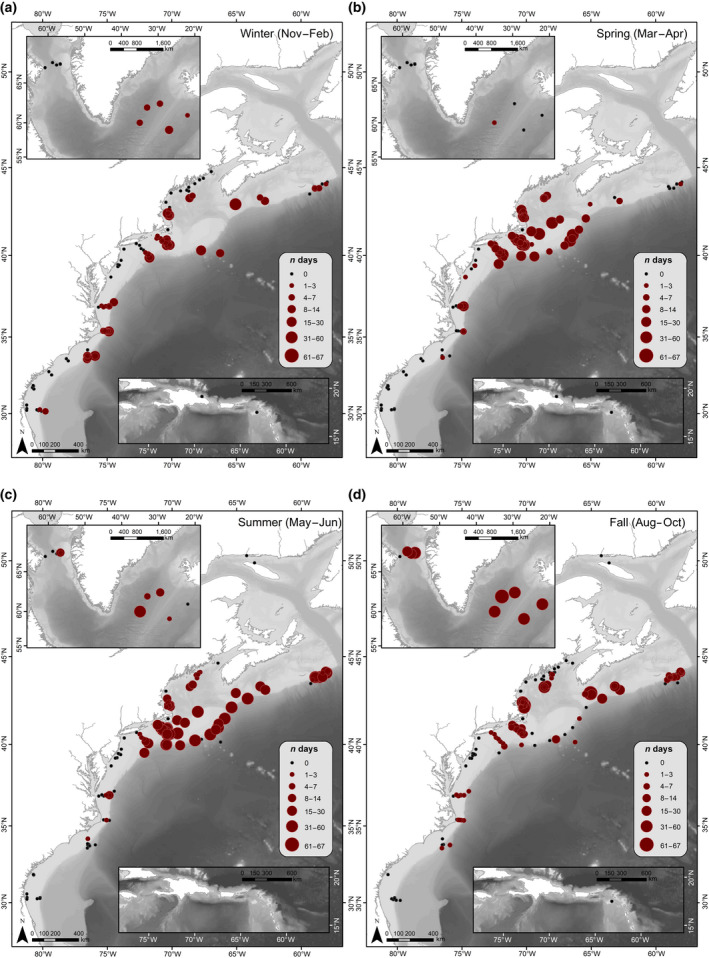
Sei whale seasonal occurrence maps: The number of days per season with confirmed North Atlantic sei whale acoustic detections, summarized for all available recording locations (2004–2014). Filled red circles indicate sei whale acoustic presence, and circle size indicates the number of days with sei whale acoustic detections during a season. Black dots indicate recorder locations with no sei whale acoustic presence for any year during that season (defined as: (a) Winter [November–February]; (b) Spring [March–April]; (c) Summer [May–July]; and (d) Fall [August–October])

**FIGURE 6:**
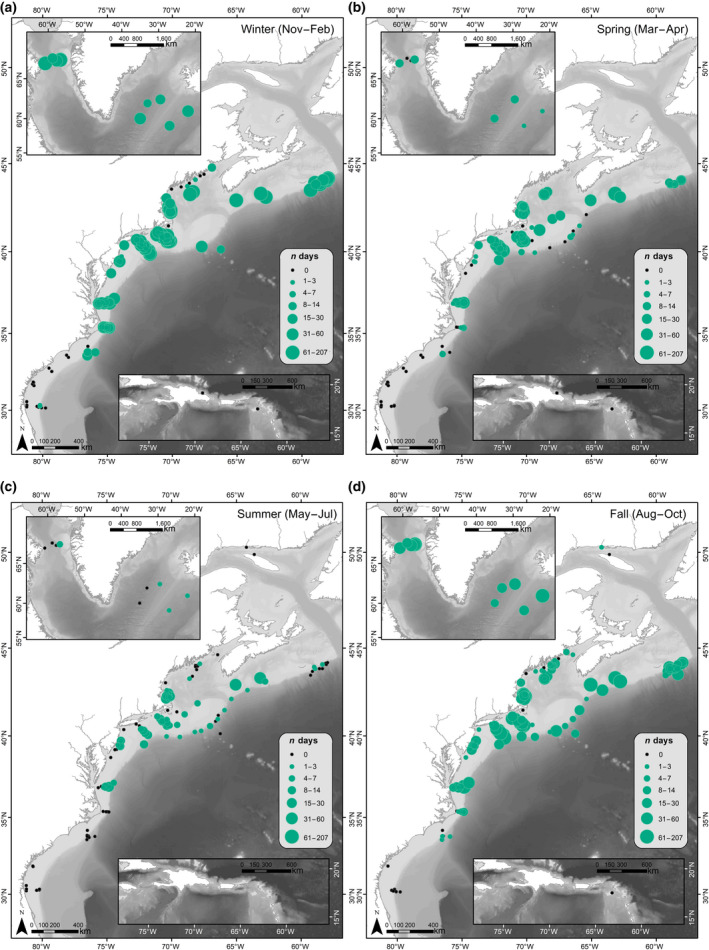
Fin whale seasonal occurrence maps: The number of days per season with confirmed North Atlantic fin whale acoustic detections, summarized for all available recording locations (2004–2014). Filled green circles indicate fin whale acoustic presence, and circle size indicates the number of days with fin whale acoustic detections during a season. Black dots indicate recorder locations with no fin whale acoustic presence for any year during that season (defined as: (a) Winter [November–February]; (b) Spring [March–April]; (c) Summer [May–July]; and (d) Fall [August–October])

**FIGURE 7:**
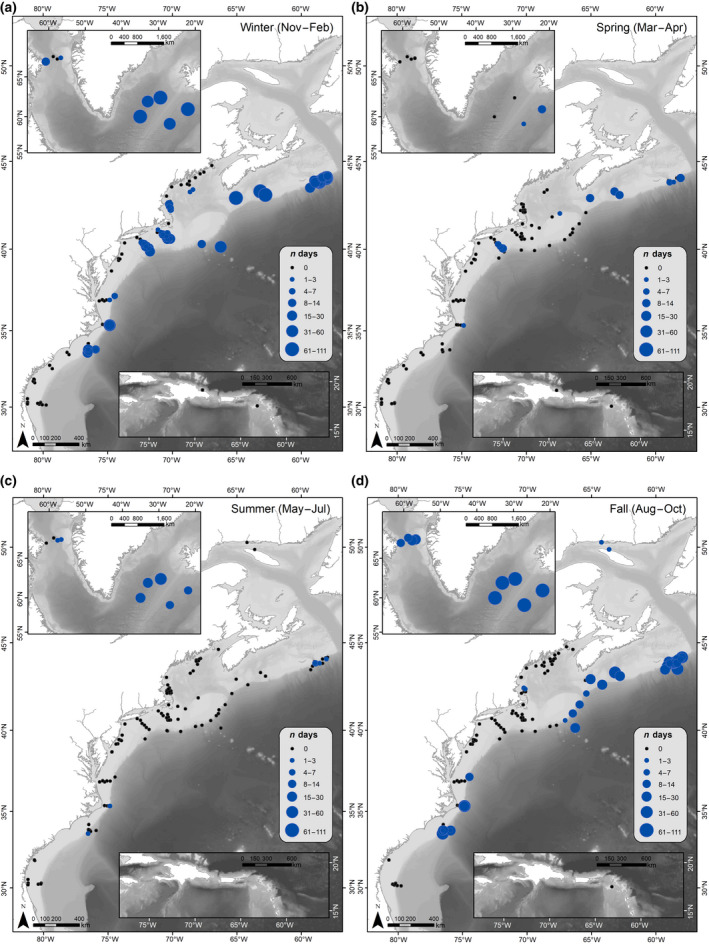
Blue whale seasonal occurrence maps: The number of days per season with confirmed North Atlantic blue whale acoustic detections, summarized for all available recording locations (2004–2014). Filled blue circles indicate blue whale acoustic presence, and circle size indicates the number of days with blue whale acoustic detections during a season. Black dots indicate recorder locations with no blue whale acoustic presence for any year during that season (defined as: (a) Winter [November–February]; (b) Spring [March–April]; (c) Summer [May–July]; and (d) Fall [August–October])

A total of 840,792 hr of recordings were processed across all available data. Acoustic detection results are presented as weekly presence for each of the corresponding vocalizations for all four species (Figure 3a–d). Each species' acoustic presence was then summarized into seasons, following the seasonality defined in Roberts et al. (2016) as: Winter (November–February); Spring (March–April); Summer (May–July); and Fall (August–October; Figures 4, 5, 6, 7). Lastly, data from Davis et al. (2017) on right whale seasonal presence was plotted together with the four species in this study to allow direct comparisons to be made between the presence of all five species (Figure S1).

### 3.1 Regional and seasonal call presence

#### 3.1.1 Humpback whales

Humpback whale songs and calls were detected on at least 1 day in all recording regions (Figures 3a and 4). They were detected year‐round in the Gulf of Maine (regions 4 and 5), southern Scotian Shelf (region 3), and off eastern Greenland (region 2). They were detected sporadically, but throughout the year, in mid‐Atlantic waters off Virginia (region 8), with the majority of humpback whale presence occurring between January and May. Humpback whales were present for a minimum of 5 days in the Davis Strait (region 1) during July and November to January. They were likely present for longer here; however, distinguishing humpback whale song from bowhead whale song in this region remains challenging.

During winter and spring months, they were detected throughout the entire sampled range, from their known Caribbean breeding grounds (region 11) through eastern Greenland (region 2; Figure 4a,b). They were detected consistently in these seasons in the northern Caribbean (January–May; region 11), but were present only for a few days in inshore waters in the Southeast United States (region 10), with only one additional day of presence in the summer, suggesting that they rarely come onto the continental shelf in this area. Winter and spring had high detection rates of humpback whales in southern New England waters (within the New York Bight to Nantucket Shoals, region 7) and in the mid‐Atlantic off Virginia (region 8).

Humpback whales were detected off Cape Hatteras (region 9) primarily between October and January, during their southward migration, with only a few detection days in spring and fall. In most summer and fall months, humpback whale detections decreased noticeably in southern New England waters (region 7), as well as eastern Greenland (region 2), reflecting concentrated humpback whale presence on feeding grounds from the Gulf of Maine to southern Scotian Shelf (regions 3–5; Figure 4c,d). The offshore recorder on the New England Seamounts (near Georges Bank; region 6) had only a few days of song and call occurrence in winter and summer (with no recording effort available in the spring). In this region (6), vocalizations were found more often on recorders along the shelf break around Georges Bank from March through July, suggesting humpback whales likely remain on the shelf, or close to it, in the northern regions.

#### 3.1.2 Sei whales

Sei whales were detected from south of Cape Hatteras to the Davis Strait (regions 1–10) and exhibited a distinct seasonal pattern in acoustic presence across the different geographic regions (Figures 3b and 5). In the Southeast United States, sei whales were detected only on recorders deployed on the western edge of Blake's Plateau (region 10), with no occurrence found on recorders closer to shore on the shelf. Sei whale calls were absent from recording areas in the Caribbean (region 11).

Sei whale calls occurred nearly year‐round in waters south of New England (region 7), with higher detection rates occurring from March through July. In the winter, sei whale acoustic detections occurred along the entire coastline, from Florida (Southeast United States; region 10) to eastern Greenland (region 2), but were sparse on recorders closest to shore, and only detected off eastern Greenland (region 2) in the beginning of November (Figure 5a).

Sei whales were detected more frequently in northern regions starting in the spring, with detections occurring primarily in waters south of New England (regions 6 and 7) and in the Gulf of Maine (regions 4 and 5; Figure 5b). The northernmost regions (Davis Strait and eastern Greenland, regions 1 and 2) had sei whale calls present starting as early as April (region 2) and June (region 1), with a majority of sei whale detections in these regions occurring from June through October. Georges Bank (region 6) had high sei whale detections from March through July, and October through December, suggestive of movements between northern and southern regions during these times.

In summer months, detections remained relatively absent south of the New York Bight (regions 8–10), with the exception of presence of 2 days off Virginia (mid‐Atlantic; region 8) and 1 day off the Southeast United States (region 10) in August and July, respectively (Figure 5c). Detections continued in these upper latitudes throughout the fall (Figure 5d), with occasional presence of sei whale calls south of New England (region 7) through Cape Hatteras (region 9), as the distribution of call occurrence expanded further south in winter months. Southbound migration was evident with detections ending in October in the Davis Strait (region 1), with a clear drop in detections over the month of October off eastern Greenland (region 2).

#### 3.1.3 Fin whales

Fin whale calls were present across the entire dataset from just south of Cape Hatteras to the Davis Strait (regions 1–10; Figures 3c and 6). Fin whale calls were present on a few Southeast US recorders (region 10); however, all detection days here were on recorders located off the continental shelf, suggesting that fin whales occurred further offshore in the south. There were no fin whale detections on any of the Caribbean (region 11) recorders, or inshore Southeast US (region 10) recorders.

Throughout the entire year, fin whales were detected near‐continuously from Virginia (mid‐Atlantic; region 8) through eastern Greenland (region 2). Of these regions (2–8), the highest number of days with detections occurred from August through April, with a noticeable decrease in days with detections from May through July.

Fin whales were detected on Georges Bank (region 6) from March to December, with sporadic presence from May to August, and no detections in January or February. Acoustic activity decreased slightly in spring months, however, fin whale detections remained present within the range (Figure 6b). From March through April, fin whales were primarily detected from the Scotian Shelf through the mid‐Atlantic (regions 3–8), with some detections in the Davis Strait (region 1), eastern Greenland (region 2), and Cape Hatteras (region 9). In summer months, acoustic activity decreased noticeably, where they were absent in the Davis Strait (region 1) from April through June and in waters south of the mid‐Atlantic (regions 9 and 10) from April (region 10) or May (region 9) through August (Figure 6c).

#### 3.1.4 Blue whales

Blue whales had the lowest number of days with detections throughout the dataset (Figure 3d). Overall, they were detected from North Carolina (Southeast United States; region 10) through the Davis Strait (region 1; Figure 7). Blue whale song did not occur on any recorders south of North Carolina (Southeast United States; region 10), suggesting that the southern edge of their range lies at the start of Blake's Plateau, or that they remain in deep waters when south of Cape Hatteras (region 9). Blue whales were not detected in the Caribbean (region 11).

Blue whale calls were present nearly year‐round off eastern Greenland (region 2). However, blue whale song was most predominant in fall and winter months, with the most detections occurring in winter (Figure 7a,d). Throughout these seasons, they occurred primarily on recorders on or near the shelf break, from North Carolina (Southeast United States; region 10) to the Davis Strait (region 1). There were some regions with detections on recorders in inshore waters; blue whales were detected sporadically in the Gulf of Maine and Massachusetts Bay (regions 4 and 5), and they were detected on nearly all recorders on the continental shelf in southern New England (region 7) in the winter (Figure 7a). Detections on Georges Bank (region 6) occurred primarily from August through December, potentially moving southward to southern New England and the New York Bight (region 7) from December through March.

Spring and summer had only occasional detections of blue whales, spanning the New York Bight (region 7) to eastern Greenland (region 2) in the spring (Figure 7b), and the Scotian Shelf (region 3) to the Davis Strait (region 1) in the summer (Figure 7c). There were a handful of days where blue whales were detected off Cape Hatteras (region 9) and the northern edge of Blake's Plateau (Southeast United States; region 10) in the summer, but these occurrences were infrequent.

### 3.2 Comparison of acoustic detections before and after 2010

**FIGURE 8:**
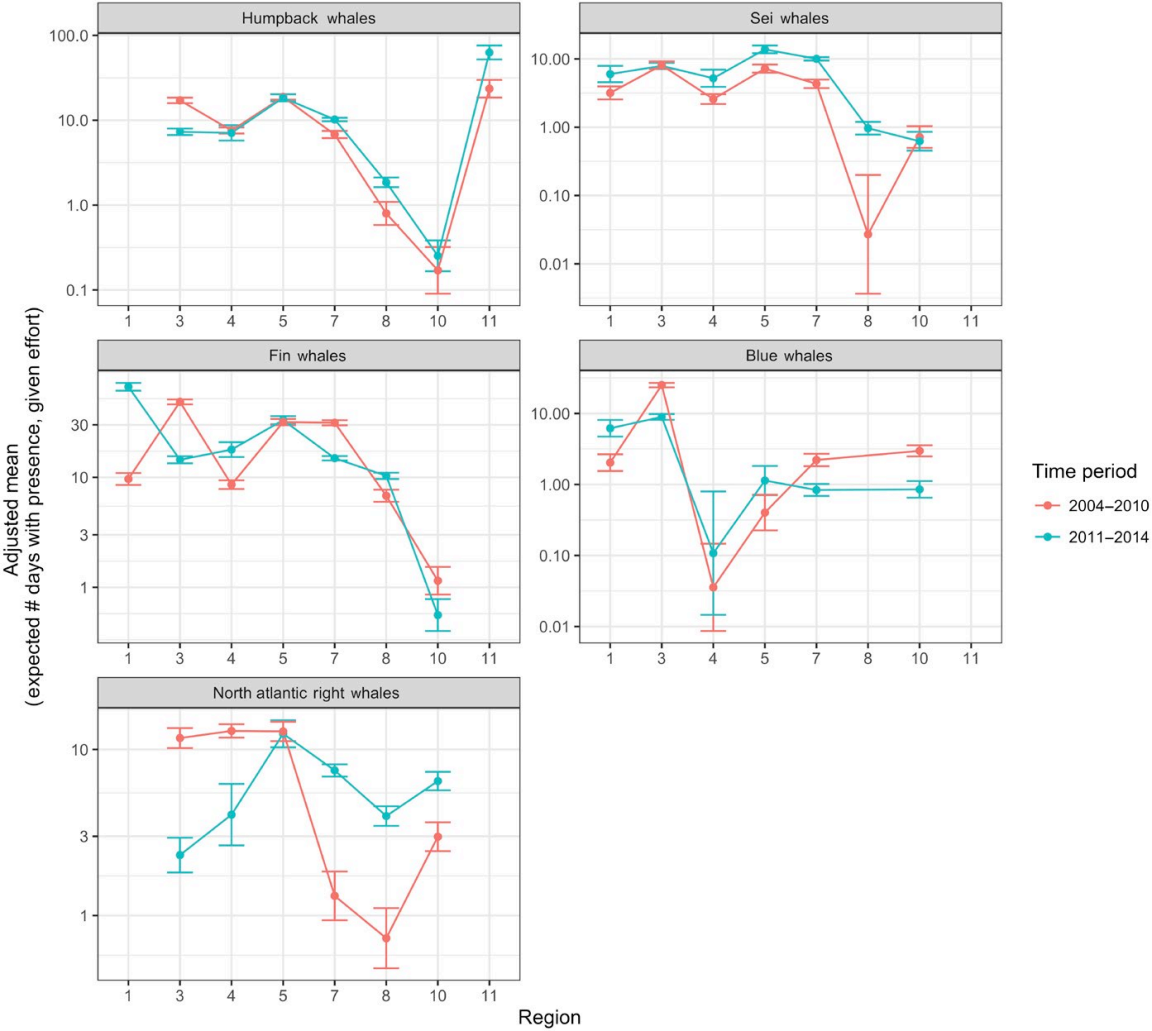
Adjusted means of acoustic occurrence for each time period (2004–2010 in red, 2011–2014 in blue), for each region indicated on the *x*‐axis, for each species. Vertical bars represent 95% confidence intervals. The *y*‐axis represents the expected number of days with acoustic presence, given the average number of recording days for that region and time period. The *y*‐axis is on a logarithmic scale (base 10) and is different for each species. Data for North Atlantic right whales are taken from Davis et al. (2017)

The annual acoustic presence before and after 2010 was evaluated for all four species, with NARWs included for comparative purposes (Figure 8). In addition, changes in weekly presence before and after 2010 are illustrated in Figure S2a–e. Of all five baleen whale species, humpback whales showed the least change before and after 2010, with only a marked decrease in acoustic presence on the Scotian Shelf area (region 3) after 2010 (Figure 8; Figure S2a; Table 3a). Sei whales had an increased acoustic presence after 2010 in all regions except the Scotian Shelf and Southeast United States (regions 3 and 10; Figure S2b; Table 3b). This increase in presence in the mid‐Atlantic regions (regions 7 and 8) is similar to that observed in NARWs. In contrast to sei whales, NARWs were not detected in the Davis Strait (region 1; Davis et al., 2017), and NARW acoustic presence decreased in the Gulf of Maine (region 4) after 2010 (Figure S2e). After 2010, sei, fin, and blue whale acoustic occurrence significantly increased in the northern waters of Davis Strait (region 1), with an increase for sei and fin whales in the Gulf of Maine (region 4; Figure S2b–d, Tables 3b–d). Like the other species, the presence of fin and blue whales decreased on the Scotian Shelf area (region 3) after 2010. In addition, fin and blue whale presence decreased after 2010 in southern New England waters (region 7), while blue whale presence also decreased in the Southeast United States (region 10). NARW detections showed significant decrease in northern regions (regions 3 and 4) and significant increase in southern regions (regions 7, 8, and 10) after 2010, which was not exhibited by any of the other species.

**TABLE 3:** Results of the Poisson generalized linear model (GLM) testing whether the annual occurrence of each species across regions differed over two time periods (A: 2004–2010; B: 2011–2014). The number of days per year in which whale calls were detected is the dependent variable, and the time periods and regions are independent variables, with their interaction effects included in the model. Eastern Greenland, Georges Bank, Cape Hatteras, and the Caribbean (regions 2, 6, 9, and 11) are excluded from the model due to insufficient data in some time*region cells, and the Caribbean (region 11) is included for humpback whales only. For all other regions, both factors and their interactions were significant. Tables show results from the Poisson GLM testing between the two time periods (A‐B) for each region separately, using the False Discovery Rate to correct for alpha‐value inflation for (a) humpback whales, (b) sei whales, (c) fin whales, and (d) blue whales. Pairwise comparisons of time periods across individual regions were run using the R Package *phia*

Region	Value	*df*	Chi‐square	*p*‐value
(a) Humpback whales				
A‐B: 1	0.000	1	0.001	.973
A‐B: 3	2.346	1	22.346	<.001
A‐B: 4	1.068	1	0.348	.741
A‐B: 5	1.023	1	0.143	.806
A‐B: 7	0.665	1	57.429	<.001
A‐B: 8	0.430	1	24.863	<.001
A‐B: 10	0.675	1	1.081	.478
A‐B: 11	0.374	1	41.693	<.001
Residuals: 72				
(b) Sei whales				
A‐B: 1	0.530	1	13.123	<.001
A‐B: 3	1.022	1	0.072	.788
A‐B: 4	0.495	1	17.775	<.001
A‐B: 5	0.524	1	47.099	<.001
A‐B: 7	0.430	1	118.505	<.001
A‐B: 8	0.028	1	12.645	<.001
A‐B: 10	1.151	1	0.340	.653
Residuals: 63				
(c) Fin whales				
A‐B: 1	0.145	1	665.017	<.001
A‐B: 3	3.360	1	717.504	<.001
A‐B: 4	0.480	1	65.986	<.001
A‐B: 5	0.960	1	0.603	.437
A‐B: 7	2.102	1	443.271	<.001
A‐B: 8	0.659	1	34.207	<.001
A‐B: 10	2.051	1	10.614	.001
Residuals: 63				
(d) Blue whales				
A‐B: 1	0.328	1	33.835	<.001
A‐B: 3	2.808	1	303.157	<.001
A‐B: 4	0.330	1	0.819	.427
A‐B: 5	0.354	1	7.766	.007
A‐B: 7	2.646	1	48.470	<.001
A‐B: 8	0.000	1	0.002	.963
A‐B: 10	3.489	1	53.628	<.001
Residuals: 63				

### 3.3 Detector evaluation of missed detection rates for all call types

The LFDCS, with an MD threshold of 3.0, missed an estimated 5% of days for humpback whales, 14% of days for sei whales, and 10% of days for fin whales. With an MD of 5.0, the LFDCS missed an estimated 10% of days for blue whales (Table 4). The number of days analyzed to evaluate the missed detection rate for each species varied, ranging from 247 to 250 days for blue and sei whales, respectively, and from 678 to 1,215 days for humpback and fin whales, respectively. The ability to manually screen for the repetitive calls of humpback and fin whales was greatly facilitated by using LTSAs compared to the less frequent vocalizations of sei and blue whales; therefore, more days were efficiently incorporated in the analysis for humpback and fin whales to provide a more robust validation. It is likely that the missed detection rate for sei whales would decrease if single downsweeps were allowed to indicate sei whales' daily presence. Additionally, the strict protocols used to define the daily presence for each species further reduced acoustic presence rates, but were necessary to increase our confidence in true presence. For all four species, these rates support the LFDCS as a good detector for determining acoustic presence. This is especially evident when the scale of this study is taken into consideration, as the missed detection rate was composed over data from different regions, depths, noise environments, and throughout full years when available. Therefore, the call detections in this study represent the minimum number of vocalizations present across the region but are likely to be a good representation of true seasonal patterns in each recording region.

**TABLE 4:** Summary from missed detection rate analysis, showing number of days with true positives (whales were found present both by detector validation and manual screening), false negatives (whales were found present in manual screening but not from detector validation), true negatives (whales were not found present by either detector validation or manual screening), and resulting missed detection rates for each species

Species	True positives	False negatives	True negatives	Missed detection rate (%)	Total days analyzed
Humpback	217	11	450	5	678
Sei	31	5	214	14	250
Fin	480	53	682	10	1,215
Blue	9	1	237	10	247

## 4 DISCUSSION

All four focal baleen whale species were present throughout, from the Southeast United States (region 10) to the Davis Strait and eastern Greenland (regions 1 and 2); humpback whales ranged further south into the Caribbean (region 11). During winter, all four species were acoustically present from the Southeast United States (region 10) up to the Davis Strait and eastern Greenland (regions 1 and 2), suggesting that they occur widely throughout the western North Atlantic Ocean during this season. In interpreting our observations, it is important to recognize the limitations within the dataset, including regional gaps in acoustic coverage, varying detection ranges across species and habitats, as well as acoustic behavior limiting portions of the populations being detected. Recording locations provided widespread, but varying, temporal and spatial coverage, with some regions that had (a) extensive temporal and spatial coverage (e.g., Massachusetts Bay and southern New England; regions 5 and7); (b) partial temporal and spatial coverage (e.g., Gulf of St. Lawrence, Scotian Shelf, Gulf of Maine, and Georges Bank; regions 3A, 3, 4, and 6); or (c) little to no coverage (shelf‐edge and off‐shelf waters). Furthermore, despite differences in vocal behavior across species, where some vocalizations change seasonally (e.g., fin whale 20 Hz pulses, song) or are thought to be produced by males only (e.g., song), or where acoustic behavior remains unknown (sei whales), we broadly detected vocalizations used by each species across the entire dataset. These data give a comprehensive overview of the minimum spatial and temporal distribution of each species, adding broad‐scale, long‐term information to our current understanding of these species, filling in significant gaps, and highlighting potential changes in acoustic occurrence over time.

### 4.1 Regional and seasonal acoustic presence

#### 4.1.1 Humpback whales

We detected humpback whale song and social sounds throughout all regions in the dataset during winter. Our observations confirm both that humpback whales vocalize throughout their entire range (Mattila et al., 1987; Vu et al., 2012), and that not all humpback whales migrate to southern breeding grounds in the winter (Brown et al., 1995), with at least some individuals remaining on northern feeding grounds such as the Gulf of Maine (regions 4 and 5) and the Scotian Shelf (region 3) throughout this period (Kowarski et al., 2018). This winter distribution is not surprising, as various studies have observed humpback whales in northern latitudes throughout the year (Clapham et al., 1993; Murray et al., 2014). However, the length of time over which they were present across all areas during winter months in this study was extensive. In addition to the expected detections in the Caribbean (Heenehan et al., 2019; Whitehead & Moore, 1982), humpback whales were present from Cape Hatteras (region 9) to eastern Greenland (region 2) throughout the winter. Detections also remained high across these regions through spring and summer. Additionally, detections showed that the regions south of New England (region 7) and east of Greenland (region 2) were also important areas for humpback whales, similar to NARWs (Davis et al., 2017; Mellinger et al., 2011; Muirhead et al., 2018).

The noticeable decrease in acoustic activity across all available recorders in the fall (Figure 4), as well as recorders on the shelf in the Southeast United States (region 10) in winter through summer months, supports previous studies that suggested migration to and from Caribbean breeding grounds occurs further offshore, beyond the detection range of the recorders used in our study (Clapham & Mattila, 1990; Kennedy et al., 2014; Reeves, Smith, Josephson, Clapham, & Woolmer, 2004). The few detections (33 days) in the Southeast United States (region 10) in late winter and spring suggest that some individuals may travel through or linger in coastal waters, but this is likely an exception rather than the norm. However, decreases in humpback whale detections could also be attributed to changes in vocal behavior, where more sporadic calling could lead to missed or insufficient calls within our defined presence analysis window.

#### 4.1.2 Sei whales

Sei whales exhibited distinct seasonal movements, with peak occurrence in northern latitudes (regions 1 and 2) during late summer and fall months. Like the other species, sei whales were detected along almost the entire coast in winter months, from Florida (Southeast United States.; region 10) to eastern Greenland (region 2). In the Southeast United States (region 10), sei whales were not detected on recorders closer to shore than the western edge of Blake's Plateau, indicating a more offshore distribution in this southern area. Sei whales moved into more northern regions, the Davis Strait and eastern Greenland (regions 1 and 2), in summer months, while still occurring south to the New York Bight (region 7). Very little information existed on sei whale distribution prior to this study, with most knowledge coming from whaling records off northern Labrador and the eastern North Atlantic (Jonsgård, 1966; Mead, 1977; Prieto, Janiger, Silva, Waring, & Gonçalves, 2012). This could be due to their use of offshore, pelagic habitats (Hain et al., 1985), or the fact that sei whales can be difficult to distinguish from Bryde's or fin whales in visual surveys. Their summer occurrence near Greenland (regions 1 and 2) matches the movements of satellite tagged sei whales traveling towards the Labrador Sea in May and June (Olsen et al., 2009; Prieto et al., 2014). Acoustic occurrence of sei whales corresponded with the timing reported in previous acoustic studies, with sei whales present in the Great South Channel (Georges Bank; region 6) throughout May (Baumgartner & Fratantoni, 2008; Baumgartner, Lysiak, Schuman, Urban‐Rich, & Wenzel, 2011; Baumgartner et al., 2008), and in Massachusetts Bay (region 5) from September to November (Tremblay et al., 2019). Their occurrence along the shelf edge, particularly in Canadian and Northeast US waters (Scotian Shelf and Georges Bank; regions 3 and 6) corresponds with previous reports, however, detections occurring on the shelf in the Gulf of Maine and southern New England (regions 4, 5, and 7) highlight greater use of on‐shelf areas here than previously described (COSEWIC, 2003).

This study provides the first comprehensive analysis of sei whale distribution throughout the western North Atlantic Ocean, highlighting movements and important habitat for this species. Their movement northward in summer months suggests that their summer feeding grounds range from the Gulf Maine through the Scotian Shelf (regions 3–5). Similarly, sei whales are also detected in the summer and fall from eastern Greenland to the Davis Strait (regions 1 and 2), although it is unclear if this is one continuous population from the Gulf of Maine to the Davis Strait (regions 1–5; Prieto et al., 2014). Southern New England and the New York Bight (region 7) are highlighted as an important area for sei whales, as this is the one region where they were detected persistently year‐round. This area is an important region for baleen whale species in general, and in particular for NARWs who target the same prey as sei whales, specifically *C. finmarchicus* (Baumgartner & Fratantoni, 2008; Baumgartner et al., 2011).

#### 4.1.3 Fin whales

Fin whales were present nearly year‐round from Virginia (mid‐Atlantic; region 8) to eastern Greenland (region 2). These findings correspond with regional studies where fin whales were detected on 99%–100% of recording days in Massachusetts Bay (region 5) and the New York Bight (region 7; Morano et al., 2012; Muirhead et al., 2018). Moreover, these data reflect previous findings of year‐round fin whale presence, and support suggestions that, as in other baleen whales, not all fin whales migrate. Edwards et al. (2015) indicate that fin whales are present in high and low latitudes throughout all seasons, and our observations corroborate this observation.

The lack of fin whale detections in the Davis Strait, Cape Hatteras, and the Southeast United States (regions 1, 9, and 10) in late spring and early summer could signify movements of individuals out of these regions. In the northernmost regions (1 and 2), the increase in noise from seismic and vessel activity as sea ice retreats from its maximum extent in March may play a role in the decrease in detections during this time by masking their low‐frequency vocalizations (Klinck et al., 2012). Other possibilities for the decreased detection rates include altered acoustic behavior by singing males during this time of the year (Watkins, 1981) or possible movement of fin whales farther offshore into deeper waters, beyond the detection range of these recorders. However, the latter seems unlikely since recorders deployed near the Mid‐Atlantic Ridge observed similar seasonal occurrence of fin whales as this study, with detections occurring largely from September to April (Nieukirk et al., 2004, 2012), illustrating the large range that fin whales occupy for most of the year. Visual survey data reflect similar distributions of fin whales to those observed in our study during all seasons on the shelf from Cape Hatteras (region 9) through the Gulf of Maine (region 4), and then occurring from Cape Hatteras (region 9) to the Davis Strait and eastern Greenland (regions 1 and 2) in all seasons except March–May (Edwards et al., 2015). Overall, these data confirm much of the evidence that fin whales occupy a large portion of the shelf for most of the year (Hain et al., 1992).

#### 4.1.4 Blue whales

For a typically oceanic and rare species, blue whales were detected in the dataset on the continental shelf far more than expected. Although blue whale's song travels large distances (see Table 2), it is unlikely that all detections in our data were from individuals far offshore, as sound attenuates rapidly for the recorders in shallow shelf areas, and the presence of blue whales has been corroborated with visual sightings in many of the areas where they were acoustically detected (National Marine Fisheries Service, 2018; Wenzel et al., 1988). Blue whales were detected on the shelf north of the New York Bight (region 7), while all other detections, as far south as North Carolina (Southeast United States; region 10), were only along the shelf break. Their presence nearly year‐round from the Scotian Shelf (region 3) to eastern Greenland (region 2) supports previous acoustic and visual surveys that identified these areas as important blue whale habitats (Hooker, Whitehead, & Gowans, 1999; Marotte, 2014; Moors‐Murphy et al., 2019; Whitehead, 2013). Our results also confirm previous studies indicating the shelf break and canyons as important areas for blue whales (Moors‐Murphy, 2014).

Blue whale acoustic presence is sparse across the entire dataset from April to August. While this species' distribution likely extends beyond the recorders' range during this time, it is also likely blue whales have different acoustic behavior during these months, as shown by Moors‐Murphy et al. (2019) in Canadian waters. This study uses blue whale song to determine the presence, as it is the most common blue whale vocalization throughout the year compared to other blue whale vocalizations (Berchok et al., 2006; Marotte, 2014; Mellinger & Clark, 2003). Like other baleen whales, blue whale song is thought to be produced by males, as a reproductive display (Oleson et al., 2007). Therefore, this study represents a minimum presence of blue whales, as we are capturing only a portion of the population (reproductively active males) as they pass through these areas. Incorporating other known call types, such as D/arch feeding calls, would provide a broader understanding of blue whale's acoustic presence throughout the year, especially in areas where these calls are seasonally prevalent, such as eastern Greenland (region 2; Boisseau, Gillespie, Leaper, & Moscrop, 2008). As in the case with fin whales, increased anthropogenic noise, which overlaps with blue whales' vocalization range, could further hinder our detectability for blue whales during summer months, especially in northern regions as polar ice retreats (Klinck et al., 2012).

#### 4.1.5 Baleen whale occurrence before and after 2010

All baleen whale species showed significant changes in their acoustic occurrence between the two time periods considered in this study: before and after 2010. In particular, sei whales showed an increased presence in southern New England and mid‐Atlantic regions (regions 7 and 8), similar to that reported for NARWs (Davis et al., 2017). As both species are copepod feeders, sei and NARWs can often be found feeding together in some habitats (Baumgartner et al., 2011). Shifts in prey distribution in this part of the North Atlantic are already being reported, and are projected to increase with warming sea temperatures (Chust et al., 2014; McHenry, Welch, Lester, & Saba, 2019; Morley et al., 2018; Perry, Low, Ellis, & Reynolds, 2005), influencing baleen whale distribution. The Gulf of Maine (region 4) is one of the fastest warming ocean areas (Pershing et al., 2015). These climatological changes may help to explain the observed shift in NARW distribution after 2010 (Record et al., 2019). However, despite similar changes in occurrence in southern New England and mid‐Atlantic regions (regions 7 and 8), this study shows a marked difference in the way in which sei and NARWs' distributions changed after 2010 in the other regions.

Except on the Scotian Shelf and in the Southeast United States (regions 3 and 10), sei whale call occurrence increased after 2010 in most areas (Figure 8; Figure S2b). This contrasts with the dramatic changes in the presence of NARWs observed through visual and acoustic surveys, particularly in the Gulf of Maine (region 4). While some of the reduction in the presence of NARWs is due to the species' decline in recent years (Pace, Corkeron, & Kraus, 2017), their large‐scale distributional changes cannot be explained by the decline alone (Davies et al., 2019). These differences between sei and NARWs could be due to the differences in feeding strategies. NARWs are ram feeders, targeting extremely dense patches of *C. finmarchicus* with open mouths (Kenney, Hyman, Owen, Scott, & Winn, 1986; Kenney, Mayo, & Winn, 2001), and sei whales can gulp feed, targeting additional prey such as euphausiids or fish by skimming as they swim (Baumgartner & Fratantoni, 2008; Flinn, Trites, Gregr, & Perry, 2002; Laidre et al., 2010). Additionally, warming and melting of arctic sea ice have shown increases in shelf‐associated copepods in the North Atlantic, including early copepodid stages of *C. finmarchicus*, but decreases in abundance of later stages of this species, primarily targeted by NARWs (Greene, Pershing, Cronin, & Ceci, 2008; Grieve, Hare, & Saba, 2017). Thus, as NARWs' distribution shifts to follow the distribution of their primary prey, sei whales may remain in these areas and alter their focal prey. Alternate feeding strategies witnessed in other species, such as humpback whales' ability to prey switch (Fleming, Clark, Calambokidis, & Barlow, 2016), offers plausible explanation as to why results varied between species and across regions.

We observed that fin, blue, and sei whales increased the time that they spent in northern latitudes after 2010. Many studies have shown poleward shifts of species with climate change, particularly in northern latitudes (McHenry et al., 2019; Perry et al., 2005; Wynn, Josey, Martin, Johns, & Yésou, 2007) and are predicted to continue shifting, especially on the North American continental shelf (Morley et al., 2018). Thus, it is possible that fin, blue, and sei whales are following prey to more northern latitudes.

Lastly, on a more regional scale, a significant shift in habitat use after 2010 can be seen in the decreased acoustic occurrence of humpback, fin, blue, and NARWs on the Scotian Shelf (region 3). What might be driving this shift remains unclear; it is possible the shift reflects changes in prey availability similar to that observed in the Gulf of Maine (Sorochan et al., 2019), but there is little data to elucidate this process. Nevertheless, the data suggest that the Scotian Shelf (region 3) has become a less preferred habitat for most baleen whales since 2010.

## 5 CONCLUSIONS

This is the first study to show spatial and temporal occurrence of humpback, fin, blue, and sei whales across the western North Atlantic Ocean over long time spans and large spatial scales and to demonstrate how these species' distributions have changed over time. These species are all protected under the US Marine Mammal Protection Act, with fin, blue, and sei whales also listed as endangered under the US Endangered Species Act. In Canada, blue and sei whales are listed as endangered under the Canadian Species at Risk Act and by the Committee on the Status of Endangered Wildlife in Canada, respectively. Anthropogenic activity, including ship strike, entanglement, and ocean noise, are the leading threats to these species (e.g., Avila, Kaschner, & Dormann, 2018; Thomas, Reeves, & Brownell, 2016). Knowing when and how each of these species frequent areas that overlap with anthropogenic activity is crucial for their conservation, which is even more challenging given their widespread winter distributions. With increasing industrial use of the western North Atlantic seaboard (Gilman et al., 2016; Government of Canada, 2017), and increased concerns around climate change (Pecl et al., 2017), there is a need for cost‐effective monitoring of whale distributions and any changes therein. Many years of traditional visual surveys from vessels and aircraft have been conducted in US and Canadian waters (Lawson & Gosselin, 2009; Palka et al., 2017). Although these surveys can derive estimates of abundance, these estimates are not precise enough to detect the changes in distribution identified in this paper. PAM is effective for monitoring large areas over years, especially in seasons when visual surveys are limited, and is particularly valuable for detecting temporal trends and changes. Current technology also includes PAM in real‐time (Baumgartner et al., 2013, 2019), which can improve our management response times and inform mitigation efforts. This study highlights the wealth of information available from retroactively analyzing datasets from a wide range of study designs and goals. Continuing these types of cross‐institutional collaborations and designing surveys with clear goals in mind can allow for a better understanding of species occurrence, and can be used to recognize large‐scale changes as they transpire.

## Supporting information

Fig S1

Fig S2

Table S1

## Data Availability

All government funded acoustic data are publicly available upon request from the data owner.
