# Supplementary material for: Exploring movement patterns and changing distributions of baleen whales in the western North Atlantic using a decade of passive acoustic data
Source: Glob Chang Biol. 2020 Jul 12;26(9):4812–40. doi: 10.1111/gcb.15191 (PMC7496396; doi:10.1111/gcb.15191)
Supplement: Supplementary file 1 — Fig S1 [file GCB-26-4812-s001.pdf]

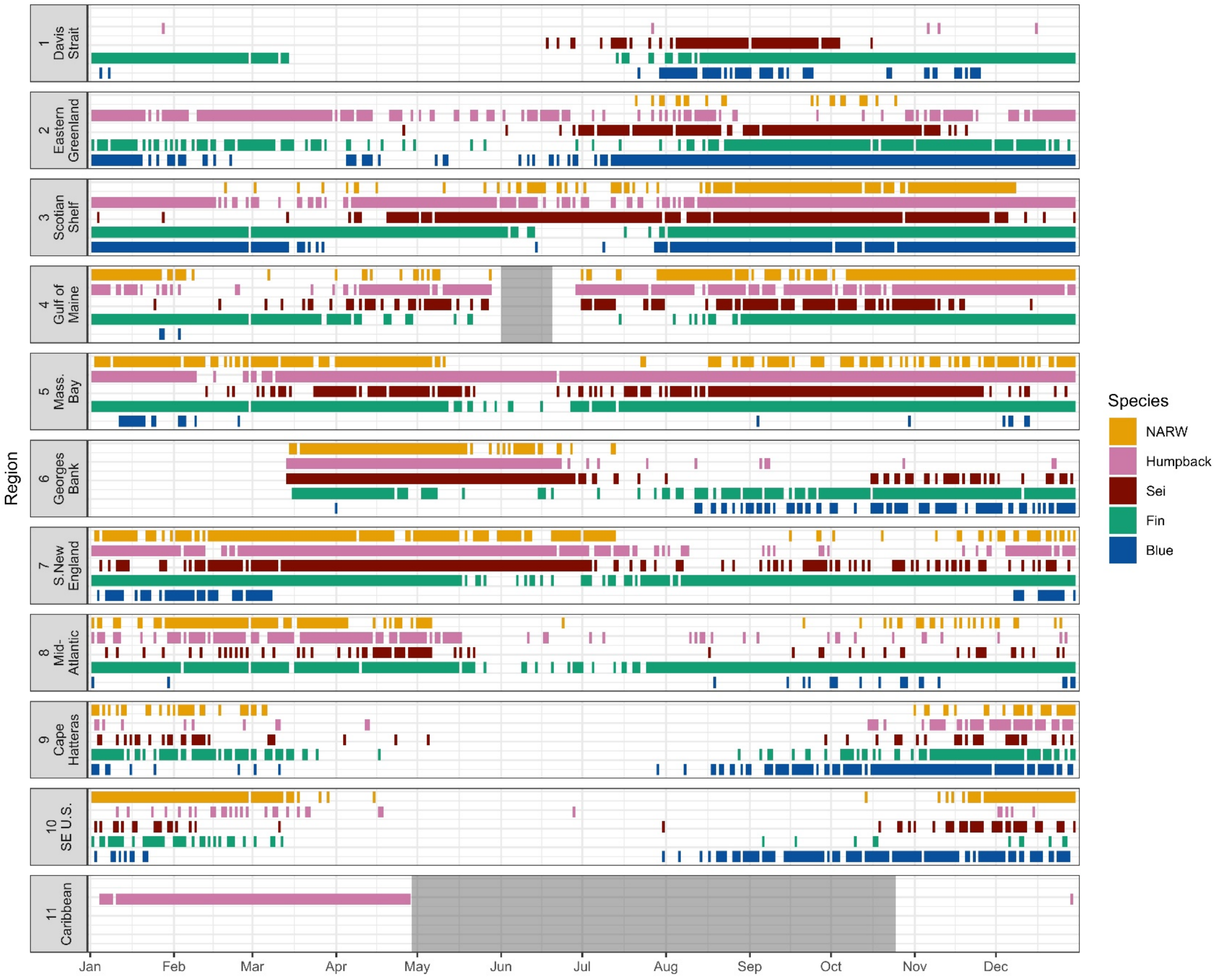

Figure S1: Daily presence summary of North Atlantic right whales (NARWs), humpback, sei, fin, and blue whales across all regions for all years of the study (2004-2014). A day of acoustic presence is indicated by orange, pink, red, green, or blue color blocks for NARW, humpback, sei, fin, or blue whales, respectively. Grey blocks indicate weeks where no data were available for that region. Daily presence of NARW is taken from Davis et al. 2017.
