# Supplementary material for: Exploring movement patterns and changing distributions of baleen whales in the western North Atlantic using a decade of passive acoustic data
Source: Glob Chang Biol. 2020 Jul 12;26(9):4812–40. doi: 10.1111/gcb.15191 (PMC7496396; doi:10.1111/gcb.15191)
Supplement: Supplementary file 2 — Fig S2 [file GCB-26-4812-s002.pdf]

(a)

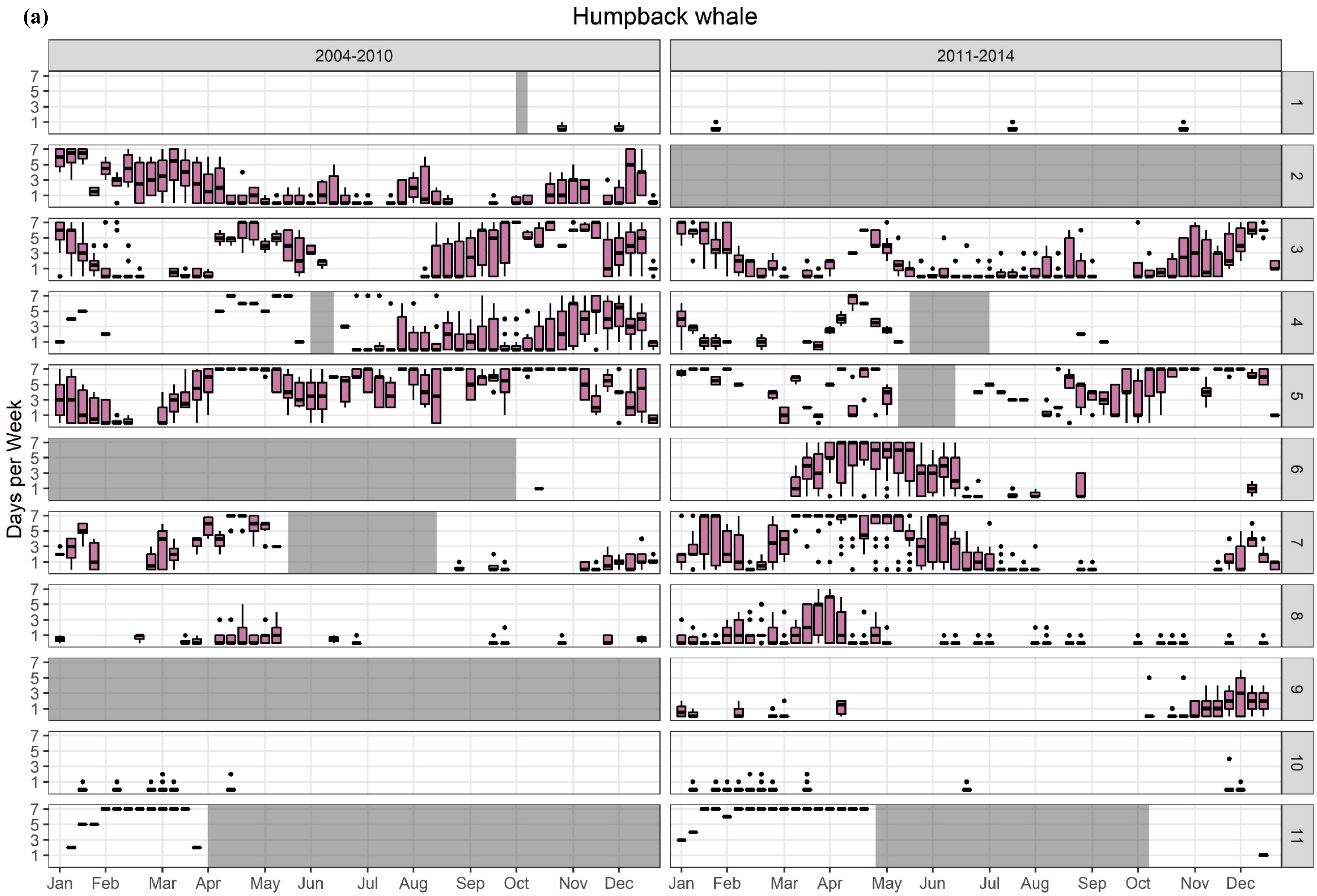

Supporting information for *Davis et al. 2020. Exploring movement patterns and changing distributions of baleen whales*.

(b)

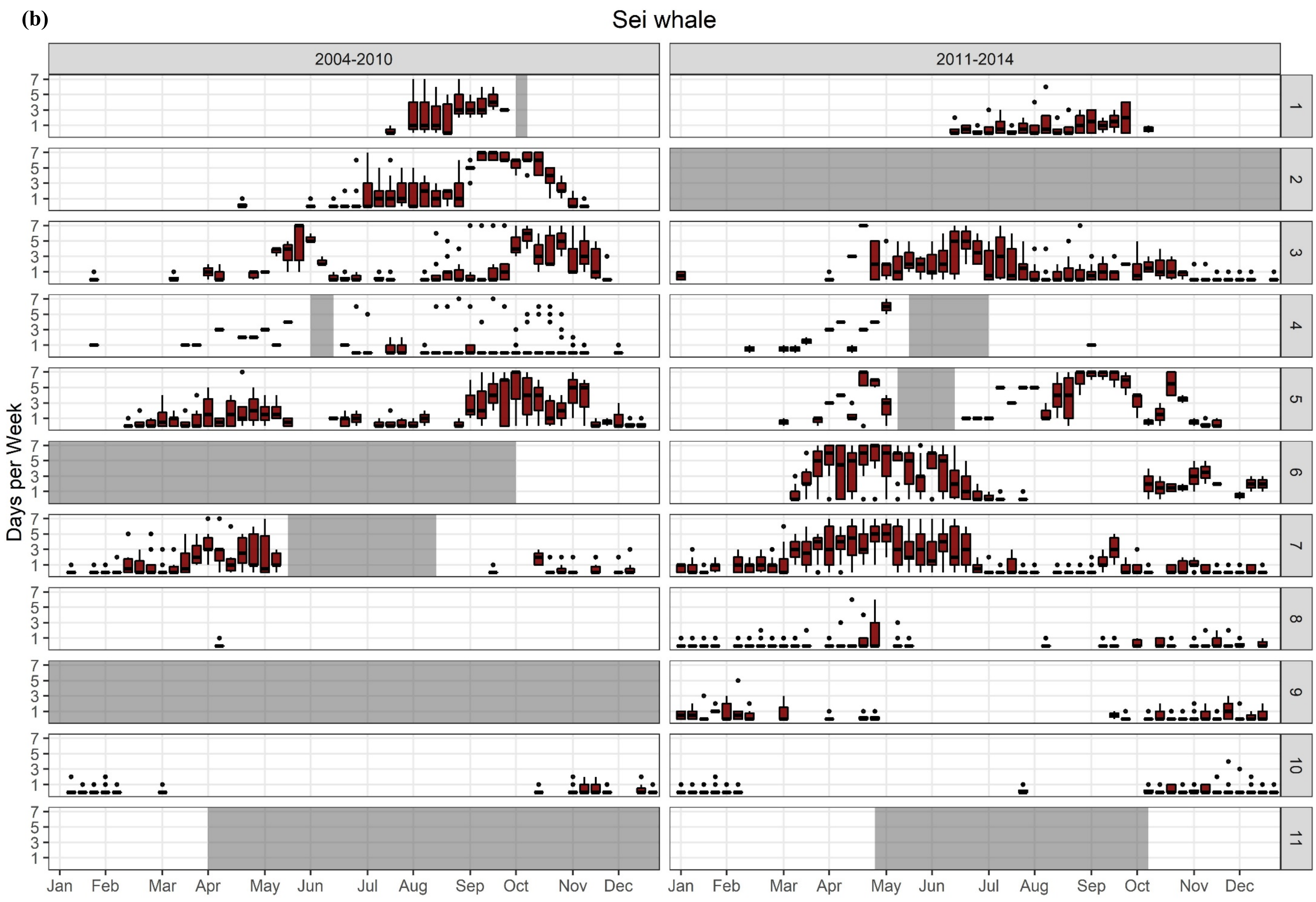

Supporting information for *Davis et al. 2020. Exploring movement patterns and changing distributions of baleen whales*.

(c)

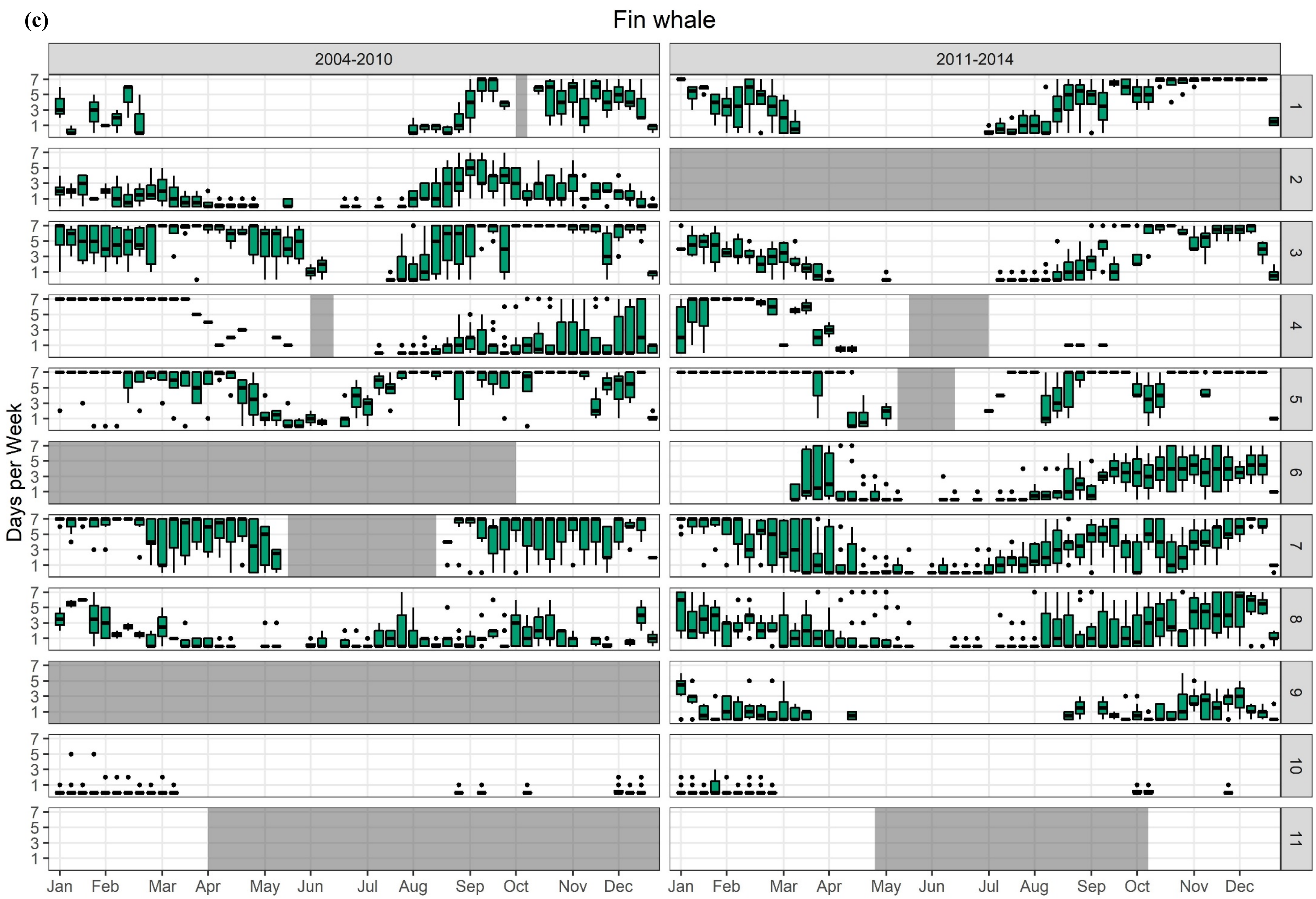

Supporting information for *Davis et al. 2020. Exploring movement patterns and changing distributions of baleen whales*.

(d)

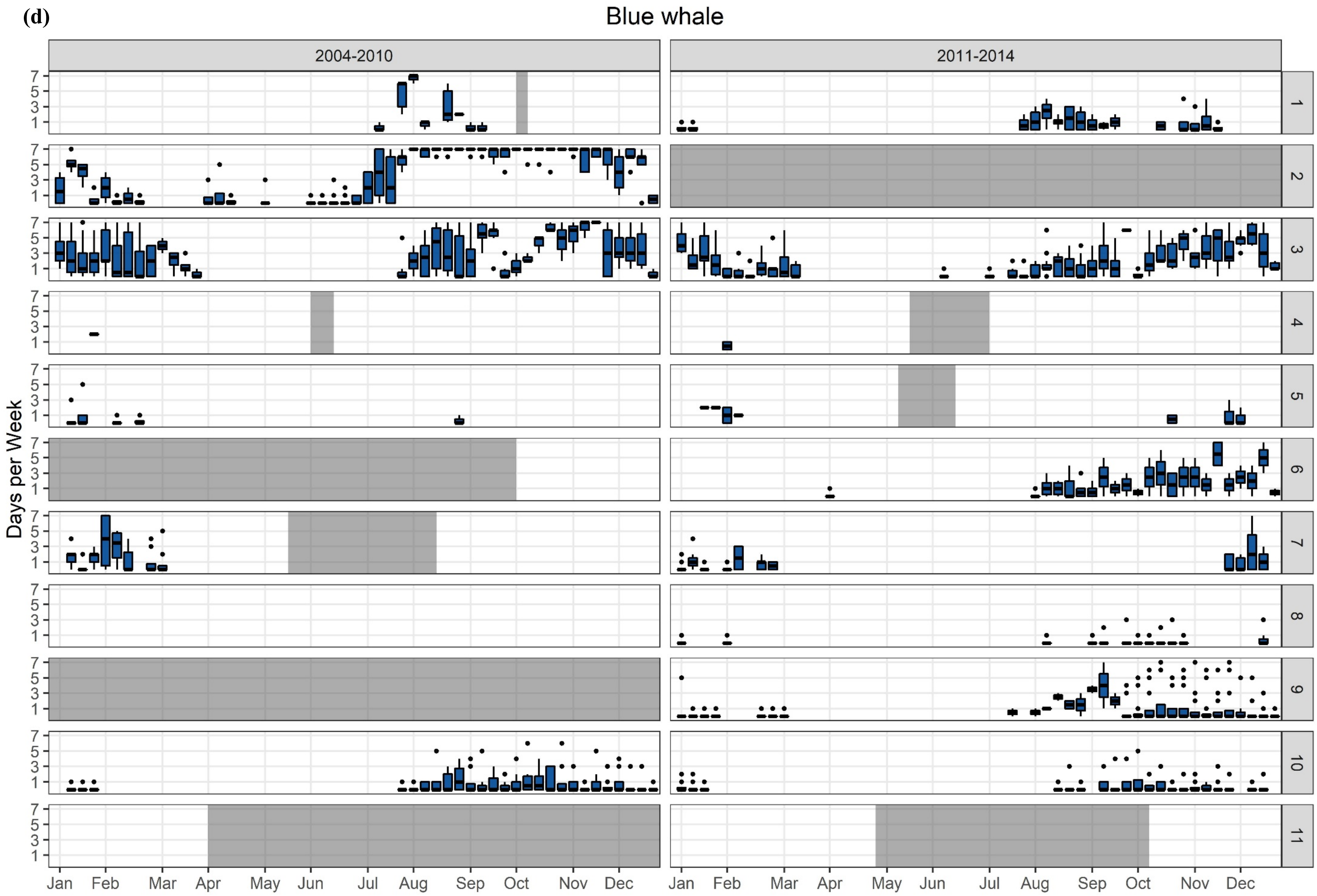

Supporting information for *Davis et al. 2020. Exploring movement patterns and changing distributions of baleen whales*.

(e)

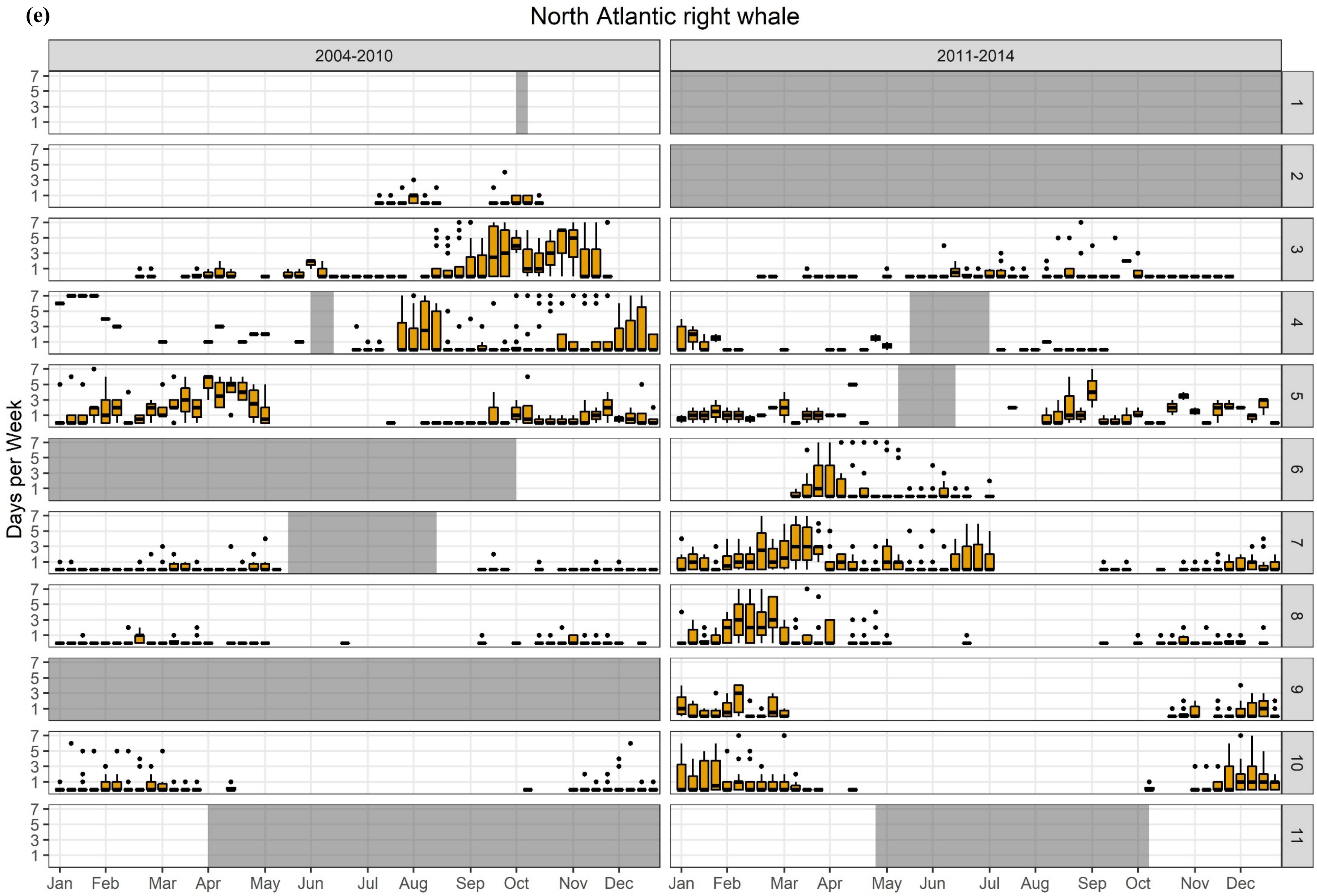

Supporting information for *Davis et al. 2020. Exploring movement patterns and changing distributions of baleen whales*.

Figure S2: Weekly Presence Comparison from 2004-2014: Boxplots representing the average number of days per calendar week with confirmed acoustic presence for **(a)** humpback whales; **(b)** sei whales; **(c)** fin whales, **(d)** blue whales, and **(e)** North Atlantic right whales (NARWs) in each region described in Figure 1 and for each time period of interest (2004-2010 and 2011-2014). Horizontal lines within the boxes indicate the median, box boundaries indicate the 25th (lower boundary) and 75th (upper boundary) percentiles, vertical lines indicate the largest (upper whisker) and smallest (lower whisker) values no further than 1.5 times the inter-quartile range, and black dots represent outliers. Grey blocks indicate time periods where no data were available for that region. Weekly presence of NARW (Figure S2e) is taken from Davis et al. 2017.

REFERENCES:

Baumgartner, M. F., and S. E. Mussoline. 2011. A generalized baleen whale call detection and classification system. Journal of the Acoustical Society of America **129**:2889-2902.

Davis, G. E., M. F. Baumgartner, J. M. Bonnell, J. Bell, C. Berchok, J. Bort Thornton, S. Brault, G. Buchanan, R. A. Charif, D. Cholewiak, C. W. Clark, P. Corkeron, J. Delarue, K. Dudzinski, L. Hatch, J. Hildebrand, L. Hodge, H. Klinck, S. Kraus, B. Martin, D. K. Mellinger, H. Moors-Murphy, S. Nieukirk, D. P. Nowacek, S. Parks, A. J. Read, A. N. Rice, D. Risch, A. Širović, M. Soldevilla, K. Stafford, J. E. Stanistreet, E. Summers, S. Todd, A. Warde, and S. M. Van Parijs. 2017. Long-term passive acoustic recordings track the changing distribution of North Atlantic right whales (*Eubalaena glacialis*) from 2004 to 2014. Scientific Reports **7**:13460.
