# Supplementary material for: Exploring movement patterns and changing distributions of baleen whales in the western North Atlantic using a decade of passive acoustic data
Source: Glob Chang Biol. 2020 Jul 12;26(9):4812–40. doi: 10.1111/gcb.15191 (PMC7496396; doi:10.1111/gcb.15191)
Supplement: Supplementary file 3 — Table S1 [file GCB-26-4812-s003.pdf]

**Supporting Information**

Davis et al. 2020. Exploring movement patterns and changing distributions of baleen whales in the western North Atlantic using a decade of passive acoustic data

*Table S1:* Summary table of the call types in each of the call libraries used from the Low Frequency Detection and Classification System (LFDCS; Baumgartner and Mussoline 2011).

| Call Library Name | Species | Call Type # | Call Type Name | Call Type Description | Number of Exemplars | Call Library Sampling Rate |
|---|---|---|---|---|---|---|
| gom9 | Sei | 1 | Downsweep | 80-30 Hz downsweep | 74 | 2 kHz |
| | | 2 | High downsweep | 130-70 Hz downsweep | 26 | 2 kHz |
| | | 3 | Long downsweep | 80-30 Hz long duration downsweep | 117 | 2 kHz |
| | NARW | 5 | Upcall | 100-200 Hz upcall | 205 | 2 kHz |
| | | 6 | Check upsweep | Attack with slight downsweep, then rapid upsweep | 144 | 2 kHz |
| | | 7 | Steep high upsweep | Rapid upsweep ending around 200 Hz | 186 | 2 kHz |
| | | 8 | Short upsweep | Short duration upsweep | 104 | 2 kHz |
| | | 9 | Long upsweep | 90-250 Hz long upsweep | 83 | 2 kHz |
| | Humpback | 15 | Upsweep | 100-150 Hz upsweep similar to NARW | 71 | 2 kHz |
| | | 16 | Tonal | 2 second tonal with slight downsweep | 31 | 2 kHz |
| | | 17 | Low-frequency downsweep | 100-150 Hz downsweep similar to sei | 181 | 2 kHz |
| | | 18 | Upsweep | 100-500 Hz rapid upsweep similar to NARW | 123 | 2 kHz |
| | | 19 | Downsweep | Long “straight” 300-100 Hz downsweep | 100 | 2 kHz |
| | | 20 | Short downsweep | Short “straight” 300-100 Hz downsweep | 123 | 2 kHz |
| | | 23 | Mid-frequency downsweep | 350-200 Hz downsweep | 192 | 2 kHz |
| | | 24 | Inverted “U” | Inverted 100-150 Hz “U” call with upper harmonic | 151 | 2 kHz |
| | | 25 | Variable downsweep arch | Variations of a 550-200 Hz downsweeping arch | 211 | 2 kHz |
| gomlf_ blue | Fin | 1 | 20-Hz pulse | Low-frequency downsweep centered at 20 Hz | 171 | 128 Hz |
| | Blue | 2 | AB call | AB Calls | 100 | 128 Hz |
| | | 3 | A call | A Call | 147 | 128 Hz |
| | | 4 | B call | B Call | 110 | 128 Hz |
