# Supplementary material for: Exploring movement patterns and changing distributions of baleen whales in the western North Atlantic using a decade of passive acoustic data
Source: Glob Chang Biol. 2020 Jul 12;26(9):4812–40. doi: 10.1111/gcb.15191 (PMC7496396; doi:10.1111/gcb.15191)
Supplement: Supplementary file 4 [file GCB-26-4812-s004.pdf]

Supporting Information

Davis et al. 2020. Exploring movement patterns and changing distributions of baleen whales in the western North Atlantic using a decade of passive acoustic data. Global Change Biology.

Table S1: Summary table of the call types in each of the call libraries used from the Low Frequency Detection and Classification System (LFDCS; Baumgartner and Mussoline 2011).

Call Library Name	Species	Call Type #	Call Type Name	Call Type Description	Number of Exemplars	Call Library Sampling Rate
gom9	Sei	1	Downsweep	80-30 Hz downsweep	74	2 kHz
		2	High downsweep	130-70 Hz downsweep	26	2 kHz
		3	Long downsweep	80-30 Hz long duration downsweep	117	2 kHz
	NARW	5	Upcall	100-200 Hz upcall	205	2 kHz
		6	Check upsweep	Attack with slight downsweep, then rapid upsweep	144	2 kHz
		7	Steep high upsweep	Rapid upsweep ending around 200 Hz	186	2 kHz
		8	Short upsweep	Short duration upsweep	104	2 kHz
		9	Long upsweep	90-250 Hz long upsweep	83	2 kHz
	Humpback	15	Upsweep	100-150 Hz upsweep similar to NARW	71	2 kHz
		16	Tonal	2 second tonal with slight downsweep	31	2 kHz
		17	Low-frequency downsweep	100-150 Hz downsweep similar to sei	181	2 kHz
		18	Upsweep	100-500 Hz rapid upsweep similar to NARW	123	2 kHz
		19	Downsweep	Long “straight” 300-100 Hz downsweep	100	2 kHz
		20	Short downsweep	Short “straight” 300-100 Hz downsweep	123	2 kHz
		23	Mid-frequency downsweep	350-200 Hz downsweep	192	2 kHz
		24	Inverted “U”	Inverted 100-150 Hz “U” call with upper harmonic	151	2 kHz
		25	Variable downsweep arch	Variations of a 550-200 Hz downsweeping arch	211	2 kHz
gomlf_ blue	Fin	1	20-Hz pulse	Low-frequency downsweep centered at 20 Hz	171	128 Hz
	Blue	2	AB call	AB Calls	100	128 Hz
		3	A call	A Call	147	128 Hz
		4	B call	B Call	110	128 Hz

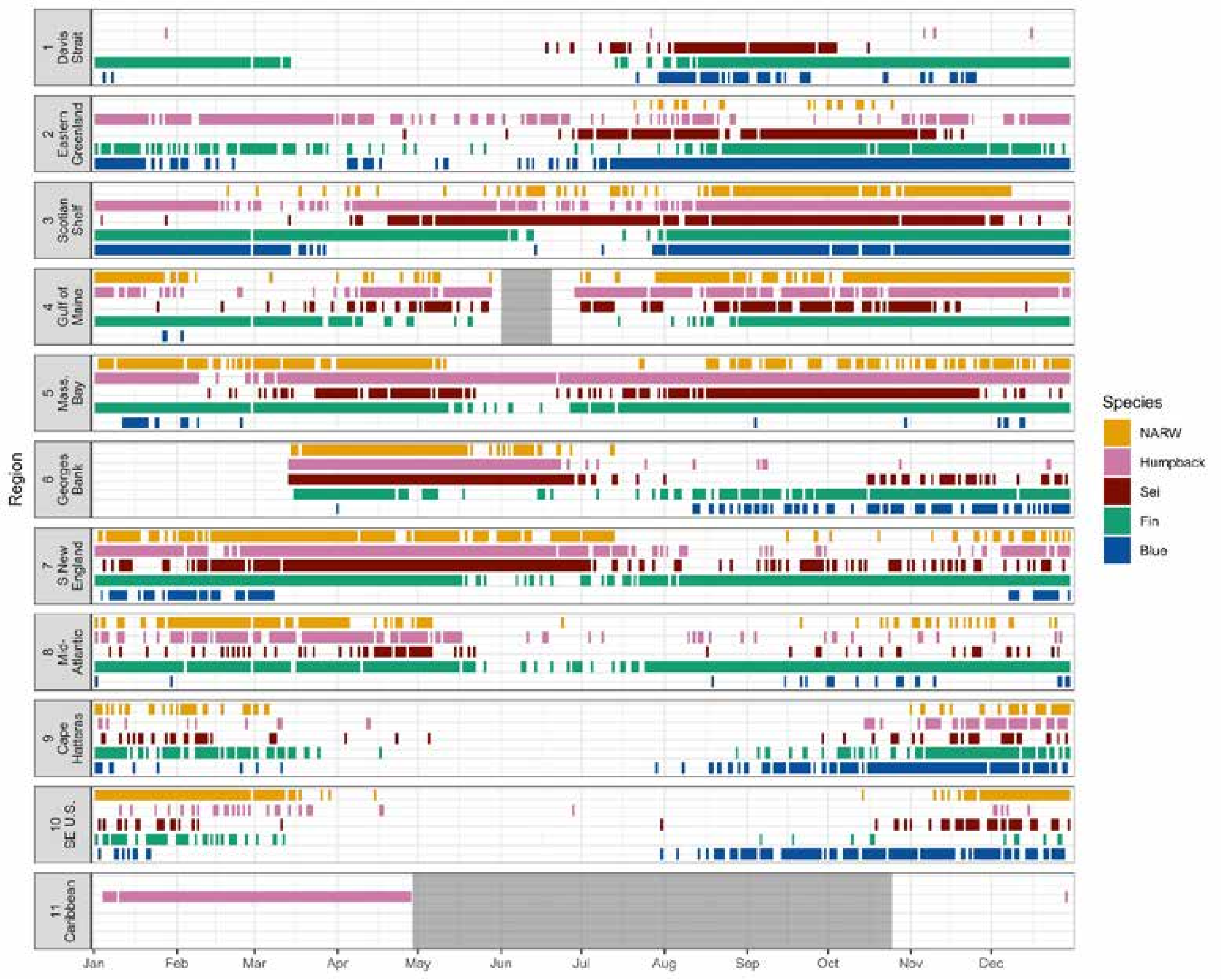

Figure S1: Daily presence summary of North Atlantic right whales (NARWs), humpback, sei, fin, and blue whales across all regions for all years of the study (2004-2014). A day of acoustic presence is indicated by orange, pink, red, green, or blue color blocks for NARW, humpback, sei, fin, or blue whales, respectively. Grey blocks indicate weeks where no data were available for that region. Daily presence of NARW is taken from Davis et al. 2017.

(a)

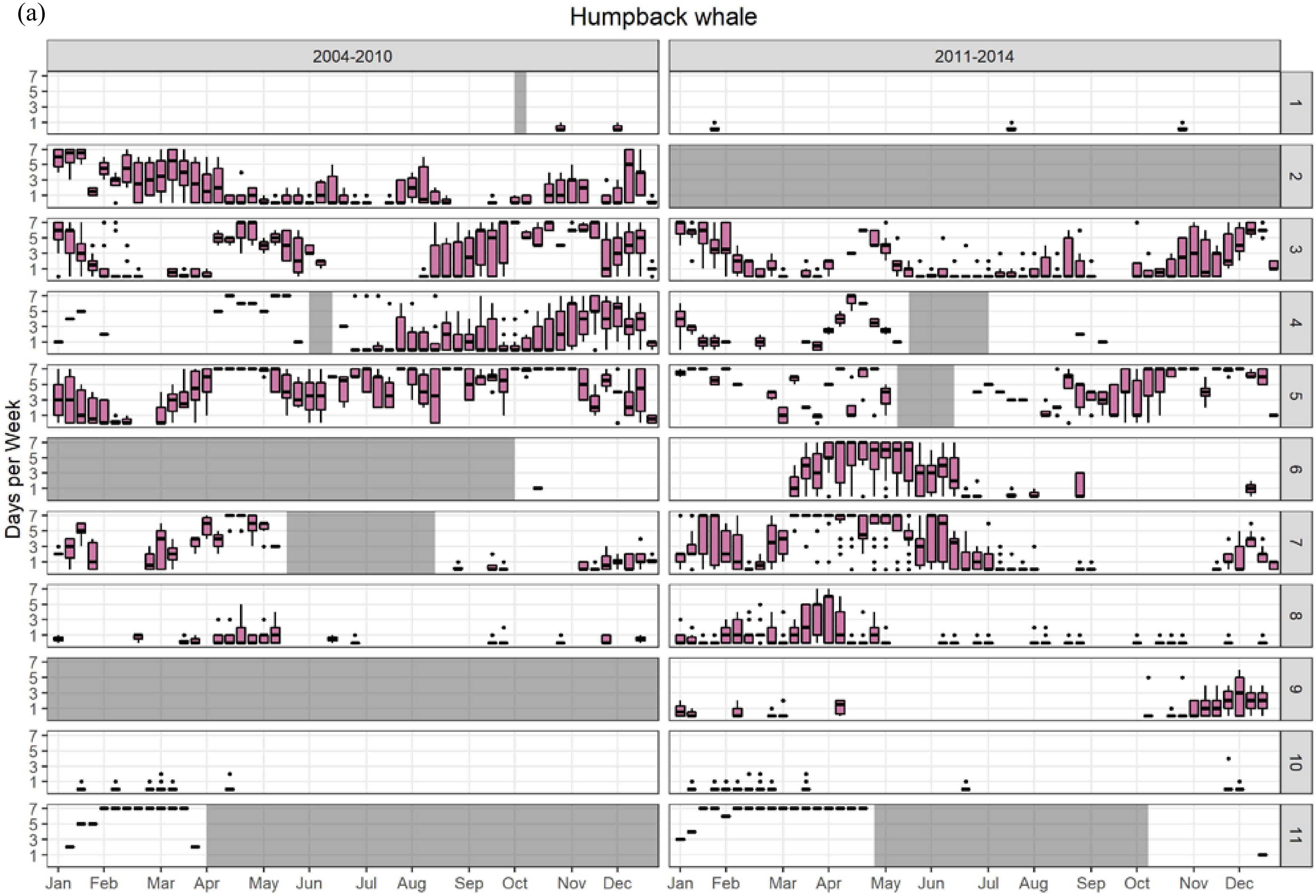

Supporting information for *Davis et al. 2020. Exploring movement patterns and changing distributions of baleen whales.*

(b)

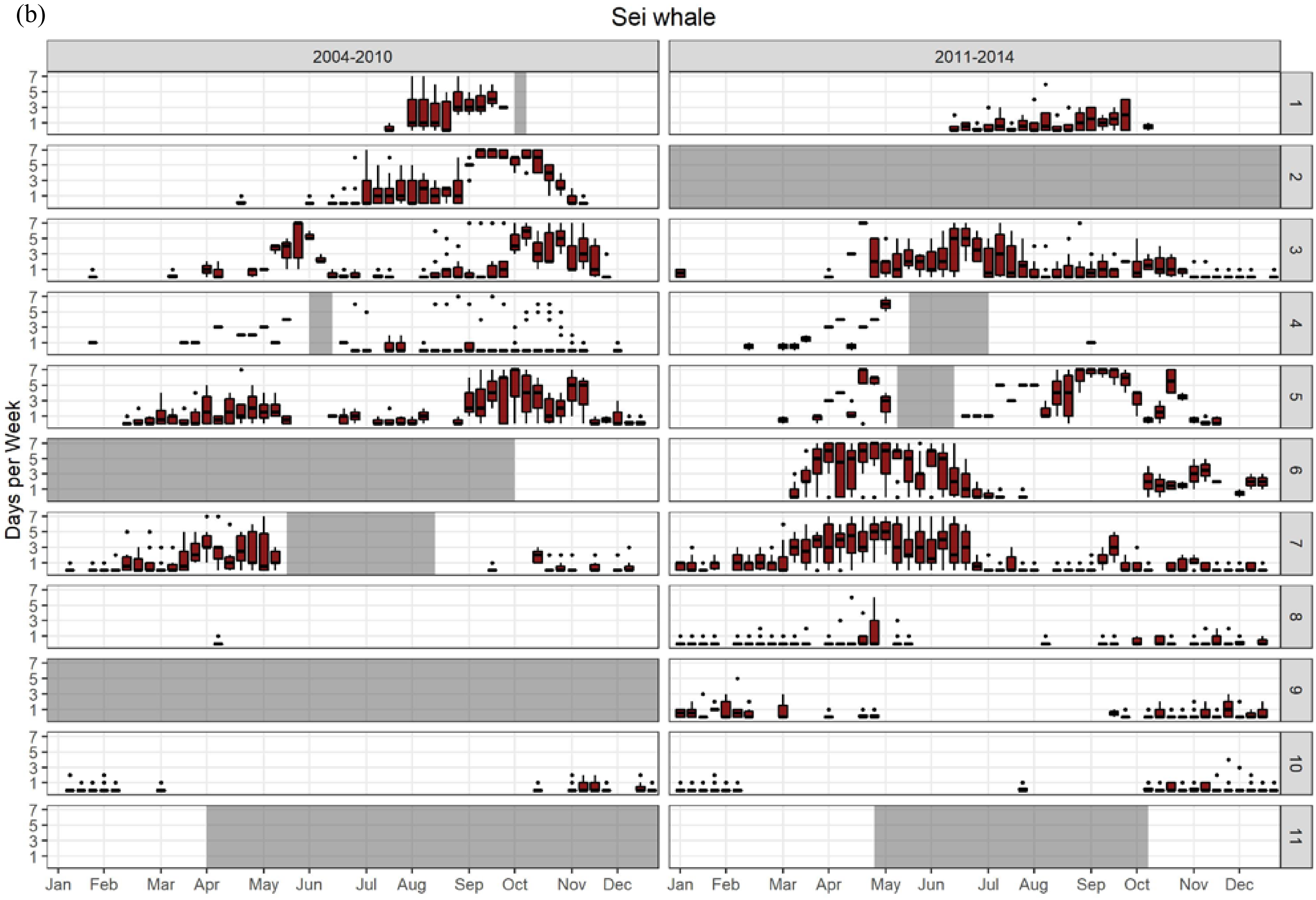

Supporting information for *Davis et al. 2020. Exploring movement patterns and changing distributions of baleen whales*.

(c)

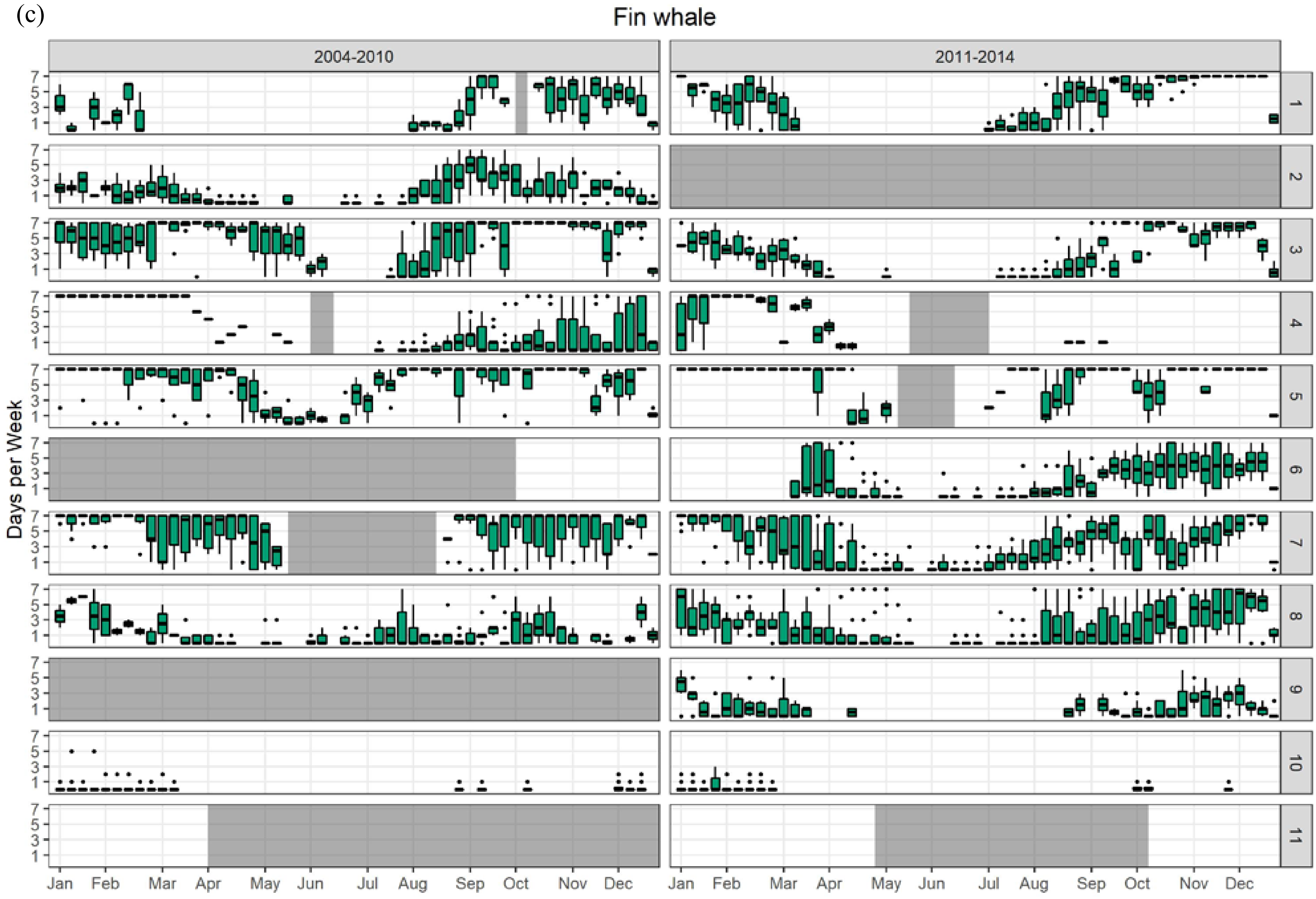

Supporting information for *Davis et al. 2020. Exploring movement patterns and changing distributions of baleen whales*.

(d)

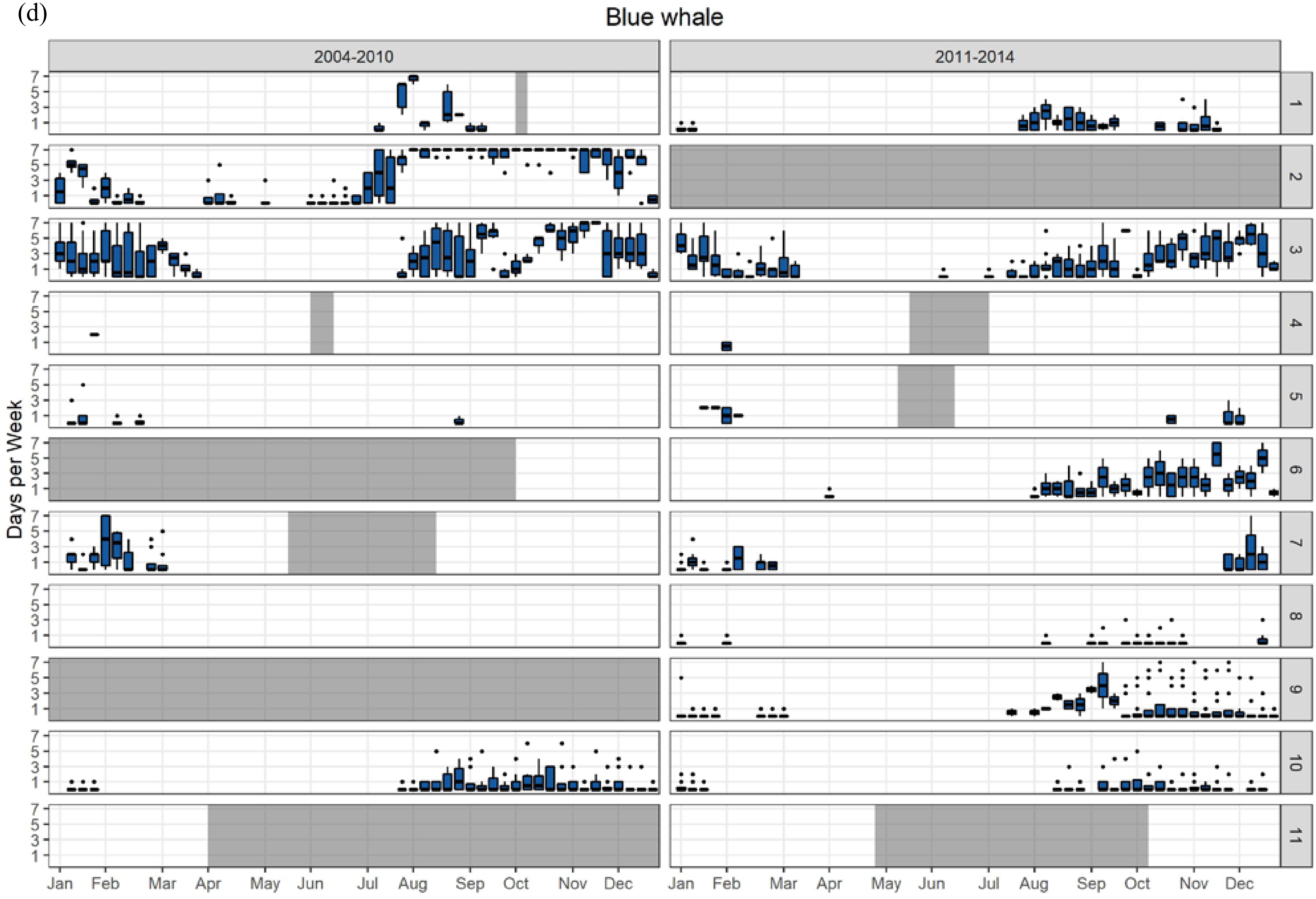

Supporting information for *Davis et al. 2020. Exploring movement patterns and changing distributions of baleen whales.*

(e)

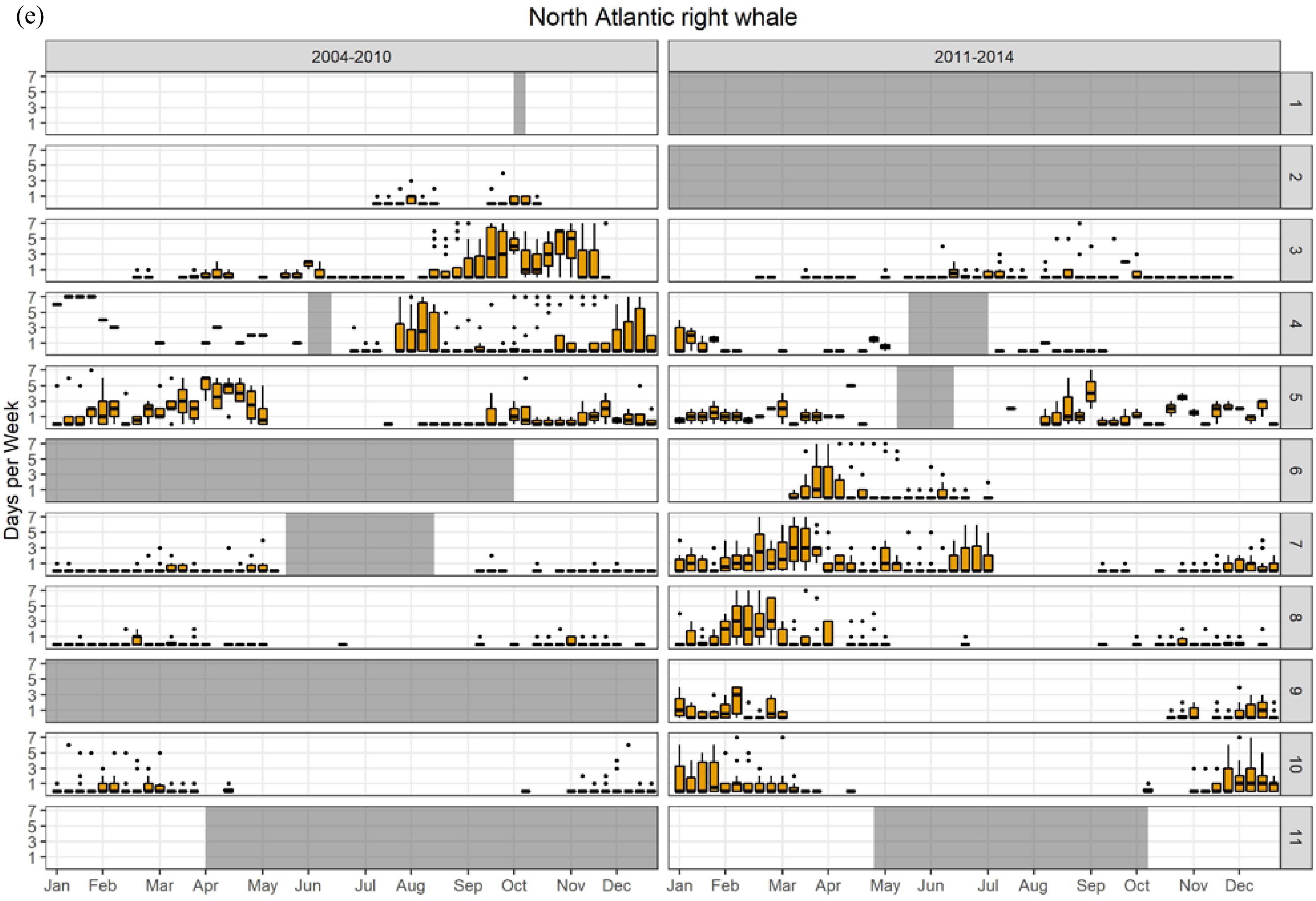

Supporting information for *Davis et al. 2020. Exploring movement patterns and changing distributions of baleen whales*.

Figure S2: Weekly Presence Comparison from 2004-2014: Boxplots representing the average number of days per calendar week with confirmed acoustic presence for **(a)** humpback whales; **(b)** sei whales; **(c)** fin whales, **(d)** blue whales, and **(e)** North Atlantic right whales (NARWs) in each region described in Figure 1 and for each time period of interest (2004-2010 and 2011-2014). Horizontal lines within the boxes indicate the median, box boundaries indicate the 25th (lower boundary) and 75th (upper boundary) percentiles, vertical lines indicate the largest (upper whisker) and smallest (lower whisker) values no further than 1.5 times the inter-quartile range, and black dots represent outliers. Grey blocks indicate time periods where no data were available for that region. Weekly presence of NARW (Figure S2e) is taken from Davis et al. 2017.

REFERENCES:

Baumgartner, M. F., and S. E. Mussoline. 2011. A generalized baleen whale call detection and classification system. Journal of the Acoustical Society of America **129**:2889-2902.

Davis, G. E., M. F. Baumgartner, J. M. Bonnell, J. Bell, C. Berchok, J. Bort Thornton, S. Brault, G. Buchanan, R. A. Charif, D. Cholewiak, C. W. Clark, P. Corkeron, J. Delarue, K. Dudzinski, L. Hatch, J. Hildebrand, L. Hodge, H. Klinck, S. Kraus, B. Martin, D. K. Mellinger, H. Moors-Murphy, S. Nieukirk, D. P. Nowacek, S. Parks, A. J. Read, A. N. Rice, D. Risch, A. Širović, M. Soldevilla, K. Stafford, J. E. Stanistreet, E. Summers, S. Todd, A. Warde, and S. M. Van Parijs. 2017. Long-term passive acoustic recordings track the changing distribution of North Atlantic right whales (*Eubalaena glacialis*) from 2004 to 2014. Scientific Reports **7**:13460.
